# Image-Based Dietary Energy and Macronutrients Estimation with ChatGPT-5: Cross-Source Evaluation Across Escalating Context Scenarios

**DOI:** 10.3390/nu17223613

**Published:** 2025-11-19

**Authors:** Marcela Rodríguez-Jiménez, Gustavo Daniel Martín-del-Campo-Becerra, Sandra Sumalla-Cano, Jorge Crespo-Álvarez, Iñaki Elio

**Affiliations:** 1 Research Group on Foods, Nutritional Biochemistry and Health, Universidad Europea del Atlántico, 39011 Santander, Spain; marcela.rodriguez@alumnos.uneatlantico.es (M.R.-J.); sandra.sumalla@uneatlantico.es (S.S.-C.); 2 German Aerospace Center (DLR), 82234 Weßling, Germany; gustavo.martindelcampobecerra@dlr.de; 3 Faculty of Health Sciences, Universidade do Cuanza, Cuito EN250, Bié, Angola; 4 Faculty of Health Sciences, Universidad de La Romana, La Romana 22000, Dominican Republic; 5 Higher Polytechnic School, Universidad Europea del Atlántico, 39011 Santander, Spain; 6 Faculty of Health Sciences, Universidad Internacional Iberoamericana, Arecibo, PR 00613, USA; 7 Department of Health, Nutrition and Sport, Universidad Internacional Iberoamericana, Campeche 24560, Mexico

**Keywords:** calorie and macronutrient estimation, image-based dietary assessment, validation metrics (MAE, MedAE, RMSE), vision-language models

## Abstract

**Background/Objectives**: Estimating energy and macronutrients from food images is clinically relevant yet challenging, and rigorous evaluation requires transparent accuracy metrics with uncertainty and clear acknowledgement of reference data limitations across heterogeneous sources. This study assessed ChatGPT-5, a general-purpose vision-language model, across four scenarios differing in the amount and type of contextual information provided, using a composite dataset to quantify accuracy for calories and macronutrients. **Methods**: A total of 195 dishes were evaluated, sourced from Allrecipes.com, the SNAPMe dataset, and Home-prepared, weighed meals. Each dish was evaluated under Case 1 (image only), Case 2 (image plus standardized non-visual descriptors), Case 3 (image plus ingredient lists with amounts), and Case 4 (replicates Case 3 but excluding the image). The primary endpoint was kcal Mean Absolute Error (MAE); secondary endpoints included Median Absolute Error (MedAE) and Root Mean Square Error (RMSE) for kcal and macronutrients (protein, carbohydrates, and lipids), all reported with 95% Confidence Intervals (CIs) via dish-level bootstrap resampling and accompanied by absolute differences (Δ) between scenarios. Inference settings were standardized to support reproducibility and variance estimation. Source stratified analyses and quartile summaries were conducted to examine heterogeneity by curation level and nutrient ranges, with additional robustness checks for error complexity relationships. **Results and Discussion**: Accuracy improved from Case 1 to Case 2 and further in Case 3 for energy and all macronutrients when summarized by MAE, MedAE, and RMSE with 95% CIs, with absolute reductions (Δ) indicating material gains as contextual information increased. In contrast to Case 3, estimation accuracy declined in Case 4, underscoring the contribution of visual cues. Gains were largest in the Home-prepared dietitian-weighed subset and smaller yet consistent for Allrecipes.com and SNAPMe, reflecting differences in reference curation and measurement fidelity across sources. Scenario-level trends were concordant across sources, and stratified and quartile analyses showed coherent patterns of decreasing absolute errors with the provision of structured non-visual information and detailed ingredient data. **Conclusions**: ChatGPT-5 can deliver practically useful calorie and macronutrient estimates from food images, particularly when augmented with standardized nonvisual descriptors and detailed ingredients, as evidenced by reductions in MAE, MedAE, and RMSE with 95% CIs across scenarios. The decline in accuracy observed when the image was omitted, despite providing detailed ingredient information, indicates that visual cues contribute meaningfully to estimation performance and that improvements are not solely attributable to arithmetic from ingredient lists. Finally, to promote generalizability, it is recommended that future studies include repeated evaluations across diverse datasets, ensure public availability of prompts and outputs, and incorporate systematic comparisons with non-artificial-intelligence baselines.

## 1. Introduction

The increasing prevalence of sedentary lifestyles and the widespread consumption of ultra-processed foods have contributed to the rising incidence of chronic diseases, including type 2 diabetes, cardiovascular disorders, and certain types of cancer [1]. Against this backdrop, accurate dietary intake assessment is key for detecting unhealthy eating patterns, guiding nutritional interventions, and supporting dietitians in both disease prevention and patient management. Therefore, there is a reinforced need for accurate and scalable dietary assessment beyond self-report methods such as 24 h recalls and food diaries, which are prone to recall bias, underreporting, and variable adherence, compromising data quality and downstream decision-making in clinical and public health practice [2,3,4].

Image-based dietary assessment offers a pragmatic alternative by leveraging meal photographs with technologies such as computer vision and, more recently, artificial intelligence (AI) [3,4,5,6,7,8,9,10]. Still, performance in real-world conditions is limited by portion size estimation, occlusion, mixed dishes, lighting variability, camera angle, and cultural diversity of foods, all of which can introduce systematic and random errors in nutrient prediction from images.

Zheng et al. [4] carried out an exploratory review of AI models employing different input modalities (text, food images, jaw motion, and multimodal data), reporting variations in accuracy depending on the input type. Shonkoff et al. [5] conducted a systematic review on the use of AI in dietary assessment through food photographs, concluding that AI-based estimations of food volume and caloric content are comparable to those of dietitians and may even surpass them. Similarly, Sultana et al. [6] performed a systematic review of deep learning (DL) approaches applied to food photographs for classification, portion size estimation, and nutrient analysis, concluding that current models still face challenges in accuracy and generalizability for real-world dietary assessment.

In parallel, several studies have proposed dedicated AI models. Fang et al. [7] introduced an energy-distribution approach; Akpa et al. [8] tested the use of reference objects to improve calorie estimation; Ege et al. [9] compared convolutional neural networks in multitask and monotask configurations; and Wang et al. [10] trained a DL model on large-scale food image datasets. Additional efforts have focused on the development of databases and algorithmic resources to strengthen reliability. Wang et al. [10] employed the Nutrition5k and ChinaMartFood109 datasets to validate novel models, while Larke et al. [11] created the Surveying Nutrient Assessment with Photographs of Meals (SNAPMe) database to support standardized evaluation. Collectively, these studies highlight the critical role of robust, well-structured, and diverse image repositories in advancing AI-driven dietary assessment.

Despite encouraging research findings, current AI models for dietary assessment based on food photographs remain largely impractical for routine use by nutrition professionals. Their implementation often requires advanced expertise in programming and machine learning, as well as access to high-performance computing resources that are typically beyond the reach of most dietitians [10,12]. Thus, the use or adaptation of these systems depends heavily on collaboration with data scientists and engineers, creating a gap between research innovation and everyday clinical practice.

Advances in vision-language models (VLMs) suggest potential to fuse visual and textual cues to improve recognition and portion reasoning, but reported that accuracy varies widely across tasks, datasets, and protocols [13,14,15]. Evaluation quality depends critically on curated references, standardized prompts/inputs, and transparent error metrics with uncertainty to enable reproducibility and fair comparison across studies and time.

Consequently, this study adopts the perspective of a dietitian-nutritionist who, despite lacking knowledge in AI, seeks to use it independently in daily practice without the need for expensive computer equipment or support from programming specialists. In this context, ChatGPT-5 [16] offers an accessible alternative, providing advanced multimodal AI through a conversational interface. Moreover, as one of the most widely recognized and publicly adopted AI systems, it aligns with the familiarity and usability required for non-technical professionals, thereby enabling practical nutritional estimation from food images. Other VLMs are currently available, such as CLIP by OpenAI [17], PaLM-E by Google DeepMind [18], and Kosmos-2 by Microsoft [19]. However, these systems require dedicated setup and Application Programming Interface (API) integration, limiting their accessibility for healthcare professionals.

Building on the experience gained from previous work in this field, this study evaluates the accuracy of ChatGPT-5 in estimating caloric and macronutrient content from food photographs, using nutritional reference data as a benchmark. The evaluation framework is defined according to key considerations from the literature, which encompass three main areas: (i) model performance, including factors that affect accuracy and strategies to improve estimation; (ii) evaluation metrics and methods for comparison with ground truth values; and (iii) database design and nutritional annotation for training and validation purposes. These are summarized in Table 1.

In line with Table 1, the present study adopts an evaluation strategy that integrates controlled scenarios with validated nutritional benchmarks, serving as the ground truth. To support the evaluation of ChatGPT-5 across diverse cultural contexts, the analysis draws on a heterogeneous set of images, including dishes prepared and annotated by dietitians, samples from SNAPMe [11], and recipe images obtained from Allrecipes.com [20]. Four case studies are addressed: Case 1 includes only the meal image, while the next two cases progressively incorporate supplementary contextual information. Case 4 uses only detailed ingredient information, omitting the corresponding image, to assess the contribution of visual input to model performance. In addition, this work analyses the relationship between the number of ingredients, as a measure of dish complexity, and the absolute error in kcal.

The remainder of the paper is organized as follows. Section 2 describes the materials and methods employed, Section 3 presents the results, followed by the discussion in Section 4. Finally, Section 5 concludes this work.

## 2. Materials and Methods

This section describes the organization of the reference nutritional data, which served as the ground truth for assessing the accuracy of the estimates generated by ChatGPT-5. It also outlines the evaluation metrics, visualization tools, and the correlation and inferential analyses applied. Finally, it presents the study cases considered and concludes by describing how ChatGPT-5 was queried to obtain the nutritional estimations.

### 2.1. Database

Recommendations from the literature emphasize the importance of using diverse, standardized, and well-annotated data sources to minimize potential biases [4,6,9,11]. Accordingly, a database of 195 dishes was compiled, combining images and reference nutritional information obtained from three complementary sources, described below:

- Allrecipes.com [20]: Provides caloric content and macronutrient distribution of meal recipes spanning diverse gastronomic traditions, accompanied by user-shared images that provide authentic visual representations of the dishes. From an estimated population of 51,000 dishes, a sample of 96 was drawn, meeting the criteria for a 10% margin of error at a 95% confidence level.
- SNAPMe [11]: Collection of food photographs annotated with portion sizes and nutritional values, contributed by U.S. participants. Images include a reference object to assist with accurate volume estimation. For a population of 275 dishes, a sample of 74 was selected for a 10% margin of error at a 95% confidence level.
- Home-prepared: Comprises 25 dishes prepared and carefully annotated by nutritionists, serving as a baseline for comparison. Nutritional data were obtained through direct quantity measurements and energy content analysis using Nutrix 1.0 [21] (a specialized software developed by Universidad Europea del Atlántico) and food equivalence tables, including the Mexican System of Food Equivalents (SMAE) [22] and the U.S. Department of Agriculture (USDA) FoodData Central [23]. A reference object (i.e., a ruler marked in centimeters) was placed in each photograph to support accurate volume and depth estimation. Images were captured with a mobile device (iPhone 16 Pro Max) under varying lighting conditions.

The reference datasets differ in origin and structure, which could introduce uncontrolled variability. To mitigate this, all nutritional entries were verified so that energy and macronutrient values were expressed in consistent units (kcal and grams (g)) and accurately reflected both the ingredient quantities and the portion sizes depicted in the corresponding images. When obvious inconsistencies were identified, macronutrient and energy values were adjusted using the SMAE [22] and USDA [23] food equivalence tables in combination with Nutrix 1.0 [21]. Records with incomplete or implausible nutritional information were excluded prior to analysis. All curated data were then organized into a consolidated database comprising all dishes, each including the corresponding image, detailed ingredient list, and reference nutritional values. Appendix A presents an example of the database structure, providing traceability and standardized access to the information required for model evaluation. Although some heterogeneity among datasets is unavoidable, this diversity enhances the ecological validity of the study by reflecting the variability typically observed in dietary records and real-world recipe data.

### 2.2. Evaluation Metrics

The performance of ChatGPT-5 was evaluated using a set of complementary quantitative error metrics. For all accuracy measures, 95% Confidence Intervals (CIs) were estimated using a non-parametric bootstrap with 10,000 resamples at the dish level.

The Mean Absolute Error (MAE) [10] and Median Absolute Error (MedAE) [24] quantified the magnitude of the errors between the ChatGPT-5 estimates ${\left\{{\hat{g}}_{j}\right\}}_{j=1}^{J}$ and the reference values ${\left\{g_{j}\right\}}_{j=1}^{J}$:

(1) $$MAE=\frac{1}{J}\sum_{j=1}^{J}\left|g_{j}-{\hat{g}}_{j}\right|,$$

(2) $$MedAE=median\left(\left|g_{j}-{\hat{g}}_{j}\right|\right).$$

In contrast to MAE, which averages all errors and may be disproportionately influenced by extreme values, MedAE provides a more robust measure by reducing the impact of outliers. It captures the central tendency of the estimation errors across observations, offering a more representative assessment of model performance under typical conditions.

Relative accuracy was evaluated using the Mean Absolute Percentage Error (MAPE) [10] and the Median Absolute Percentage Error (MedAPE) [24]:

(3) $$MAPE=\frac{1}{J}\sum_{j=1}^{J}\left(\left|g_{j}-{\hat{g}}_{j}\right|/g_{j}\right)\times \;100,$$

(4) $$MedAPE=median\left(\left|g_{j}-{\hat{g}}_{j}\right|/g_{j}\times 100\right).$$

Both MAPE and MedAPE express errors as a percentage of the reference value, facilitating interpretability. Nonetheless, these metrics are asymmetric, as they penalize overestimations more heavily than underestimations of the same magnitude. Similar to MedAE, MedAPE is less sensitive to outliers.

Error dispersion was quantified using the Interquartile Range (IQR) [25], which represents the spread of the middle 50% of the data and is robust to extreme values. Specifically, the IQR of Absolute Errors (IQRAE) and Absolute Percentage Errors (IQRAPE) were computed as:

(5) $$IQRAE=Q_{3}\left(\left|g_{j}-{\hat{g}}_{j}\right|\right)-Q_{1}\left(\left|g_{j}-{\hat{g}}_{j}\right|\right),$$

(6) $$IQRAPE=Q_{3}\left(\left|g_{j}-{\hat{g}}_{j}\right|/g_{j}\times 100\right)-Q_{1}\left(\left|g_{j}-{\hat{g}}_{j}\right|/g_{j}\times 100\right),$$

where $Q_{1}$ and $Q_{3}$ denote the first and third quartiles of the distributions of absolute and absolute percentage errors, respectively. These metrics help assess the consistency of the model’s performance across the dataset, highlighting whether errors are concentrated around the median or spread out over a wider range.

Lastly, the Root Mean Square Error (RMSE) was computed as [5,24]:

(7) $$RMSE=\sqrt{\frac{1}{J}\sum_{j=1}^{J}{\left(g_{j}-{\hat{g}}_{j}\right)}^{2}.}$$

This metric assigns greater weight to large estimation errors due to the squaring operation.

### 2.3. Visual Analysis

Scatter plots were employed to visually assess estimation performance, depicting ground truth values ${\left\{g_{j}\right\}}_{j=1}^{J}$ on the x-axis and corresponding ChatGPT-5 estimates ${\left\{{\hat{g}}_{j}\right\}}_{j=1}^{J}$ on the y-axis. Ideally, all points would align along the identity line y=x; any displacement from this line indicates estimation errors, with points above and below representing over- and underestimations, respectively. This visualization approach eases rapid identification of bias, dispersion, and overall agreement between estimated and reference values [10].

Boxplots of absolute errors for calories and macronutrients were used to compare estimation performance across the three data sources. These visualizations summarize both the central tendency and the dispersion of errors (expressed in kcal or g), allowing straightforward identification of variability and outliers. Each boxplot displays the median, IQR, and Tukey whiskers (1.5 × IQR) [26], while data points lying beyond the whiskers are shown as outliers.

### 2.4. Correlation Analysis

To evaluate whether dish complexity influenced estimation accuracy, the relationship between the number of ingredients and the absolute error in dietary energy estimation (kcal) was examined using Pearson’s correlation coefficient [9]:

(8) $$r=\frac{\sum \left(m_{i}-\bar{m}\right)\left(n_{i}-\bar{n}\right)}{\sqrt{\sum {\left(m_{i}-\bar{m}\right)}^{2}}\cdot \sqrt{\sum {\left(n_{i}-\bar{n}\right)}^{2}}},$$

where r denotes the coefficient, $m_{i}$ and $n_{i}$ are the observed values, and $\bar{m}$ and $\bar{n}$ represent their respective means. Values of r close to 1 indicate a strong positive linear relationship, values near −1 indicate a strong negative linear relationship, and values around 0 suggest the absence of correlation. To quantify the uncertainty associated with r, 95% CIs were computed using Fisher’s z-transformation [27].

To further characterize this relationship, a linear regression was fitted [9]:

(9) y=a + bx,

where y represents the dependent variable, x the independent variable, a the intercept and b the slope. This method allows the identification of systematic trends, assessment of predictive relationships, and detection of potential under- or overestimation patterns. A 95% CI for the regression line was included, along with a confidence ellipse representing the joint distribution of predicted and observed values. The CI quantifies the uncertainty in the slope b and intercept a estimates, while the ellipse captures the covariance structure of the data. A narrow ellipse reflects strong agreement and low dispersion, whereas a wider ellipse indicates greater variability or systematic bias.

### 2.5. Inferential Analysis of Estimation Errors

To complement the descriptive statistics (MAE and MedAE), inferential analyses were conducted to formally test whether estimation errors differed significantly among the three data sources (Allrecipes.com, SNAPMe, and Home-prepared). Since the dishes from each source are distinct (i.e., samples are independent and not paired), a non-parametric approach for independent samples was employed. The following hypotheses were evaluated:

- Null hypothesis (H_0_): There are no significant differences in estimation errors among the three data sources.
- Alternative hypothesis (H_1_): At least one data source differs significantly from the others.

The global hypothesis was tested using the Kruskal–Wallis rank-sum test [28], which evaluates whether the distributions of absolute errors differ significantly among the sources. The test statistic is given by:

(10) $$H=\frac{12}{L\left(L+1\right)}\sum_{i=1}^{I}l_{i}{\bar{R}}_{i}^{2}-3\left(L+1\right),$$

where L is the total number of observations, I is the number of groups, $l_{i}$ is the sample size of group i, and ${\bar{R}}_{i}$ is the mean rank of group i. Under the null hypothesis (no significant differences between sources), H approximately follows a $\chi^{2}$ distribution with I−1 degrees of freedom. A result of p<0.05 indicates that at least one group differs significantly from the others.

When the Kruskal–Wallis was significant, pairwise comparisons were conducted using the Mann–Whitney U test [29] to identify which specific pairs of sources differ. The test statistic is:

(11) $$U=k_{x}k_{y}+\frac{k_{x}\left(k_{x}+1\right)}{2}-R_{x},$$

where $k_{x}$ and $k_{y}$ are the sample sizes of groups X and Y, and $R_{x}$ is the sum of ranks in group X.

To account for multiple testing, Holm’s sequential correction [30] was applied in two stages: (i) within each variable, to adjust pairwise comparisons among the three data sources (Allrecipes.com, SNAPMe, and Home-prepared); and (ii) across variables (calories, proteins, lipids, and carbohydrates) within each experimental case, to control the overall family-wise error rate given that the errors are correlated. This two-level strategy provides a more stringent control of false positives and ensures that only statistically robust differences (unlikely to occur by chance) are interpreted as true differences among datasets. A result of $p_{Holm}<0.05$ indicates that the difference between the two groups is statistically significant.

To assess the magnitude of the detected differences, Cliff’s δ [31] was computed. This measure quantifies the degree of overlap between two distributions:

(12) $$\delta =\frac{\#\left(x_{i}>y_{j}\right)-\#\left(x_{i}<y_{j}\right)}{k_{x}k_{y}},$$

where $x_{i}$ and $y_{j}$ are observations from groups X and Y. Operator $\#\left(\cdot \right)$ denotes the number of pairwise comparisons in which the condition holds. Values of δ range from −1 to +1, with $\left|\delta \right|\approx 0.147,0.333,0.474$ corresponding to small, medium, and large effects, respectively.

### 2.6. Test Cases

The evaluation of ChatGPT-5 was conducted under four scenarios differing in the type and amount of information available. The assessment was performed using MAE, MedAE, MAPE, MedAPE, IQRAE, IQRAPE and RMSE, complemented with scatter plots and boxplots. These test cases were designed to identify model limitations, analyze the effect of non-visual information, and evaluate performance when complete ingredient data were available. Specifically, the following scenarios were considered:

1. Image only: performance assessment in the absence of contextual information.
2. Image with non-visual data: inclusion of characteristics that are difficult to capture visually, namely type and amount of fat, type and amount of sweetener, fat content of dairy products, and type of meat.
3. Image with detailed ingredient information: provision of complete ingredient descriptions and quantities to enable the most accurate estimation.
4. Detailed ingredient information only: replicates the conditions of Case 3, except that the image is omitted to isolate the contribution of visual input.

For each test case, the relationship between the number of ingredients and the absolute error in dietary energy estimation (kcal) was analyzed using Pearson’s correlation and linear regression. Given that dishes with more ingredients are commonly more complex, this analysis evaluated whether dish complexity affected estimation error and to what extent this effect is consistent across scenarios.

### 2.7. ChatGPT

This work used the Plus version of ChatGPT-5 (released in August 2025) to avoid the restrictions of the free tier and usage limits. This allowed uninterrupted access to the tool, including the uploading and processing of multiple food images. The data-sharing opt-out setting of the Plus account was activated so that uploaded images and data were processed only within the active session and not used for model training. According to OpenAI’s data policy, session content remains private and is not publicly shared, although it may be temporarily retained for security or compliance purposes. The exact retention period is not publicly specified.

The study focused on evaluating ChatGPT-5 under conditions representative of real-world professional use. Accordingly, all estimations were obtained through the ChatGPT web interface (chat.openai.com) rather than the API. This setup reflects the typical context of a dietitian-nutritionist without programming expertise, using accessible tools rather than specialized computational environments. As the chat user interface does not allow control over generation parameters such as temperature, the model operates in its default probabilistic mode.

Prompts were intentionally designed to remain simple and natural, resembling how a dietitian-nutritionist would typically interact with the system. Each case study involved specific prompts tailored to the task. The prompt for the first scenario was: “*Based on the image, calculate the energy content in kilocalories (kcal) and the amount of protein, fat, and carbohydrates in grams (g). Organize the information in a table, indicating amounts for each food item and totals*”. In the subsequent scenarios, this same prompt was adapted, for example, by adding contextual information. Appendix B illustrates how ChatGPT-5 was applied in each case study.

For Cases 1–3, each prompt included a single image corresponding to one dish, and the chat session was reset prior to each evaluation so that ChatGPT-5 had no prior information or contextual memory from previous interactions. This procedure made all estimations independent, without influence from preceding evaluations. Case 4 followed the same procedure but excluded the image.

While ChatGPT-5 is more powerful and flexible than earlier versions, it also tends to engage in broader interpretive reasoning, introducing inferred information not explicitly present in the visual or textual input. To mitigate this issue and promote analytical consistency, a set of explicit behavioral instructions was incorporated at the beginning of each evaluation to limit interpretation, enforce structured outputs, and encourage conservative, evidence-based responses. Specifically:

- “Act as a technical analyst. Do not make assumptions: describe only what the imagery and data shows.”
- “Provide responses in a literal and quantitative manner.”
- “Do not draw conclusions unless they are explicitly supported by the data.”
- “If ambiguity exists, state it explicitly rather than attempting to resolve it.”
- “Present intermediate calculations before the final result, and clearly indicate any estimated values.”

## 3. Results

**Figure 1:**
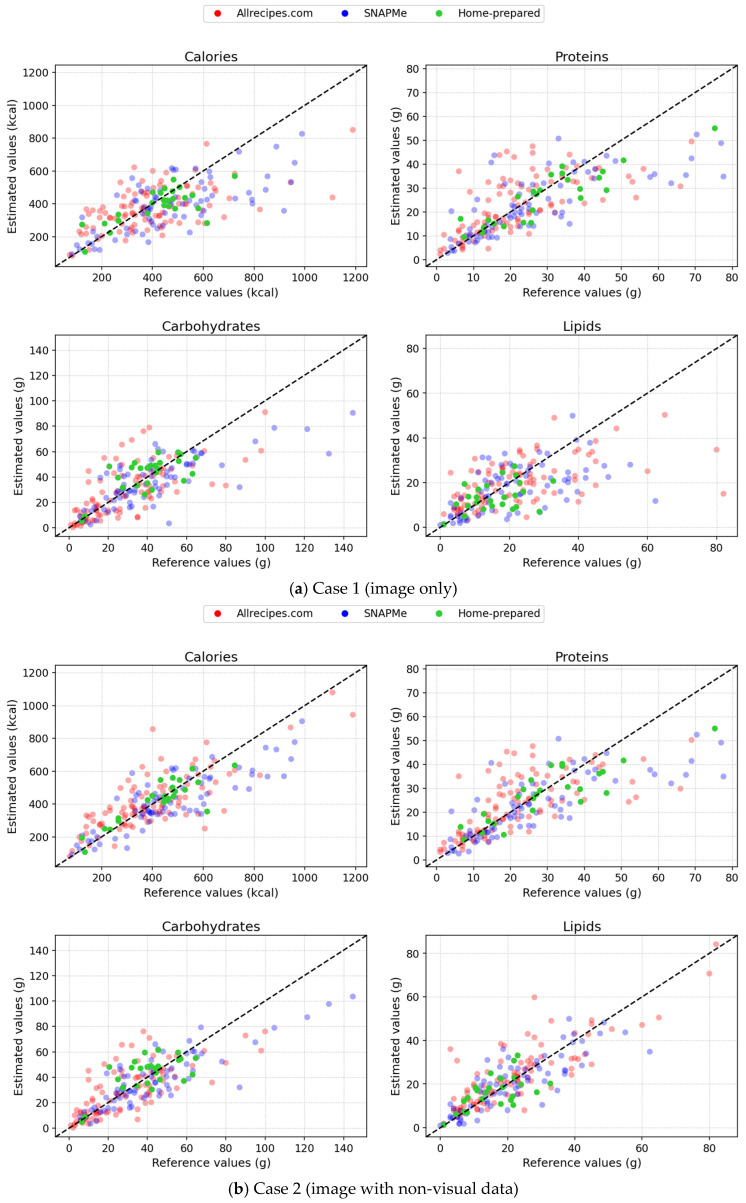

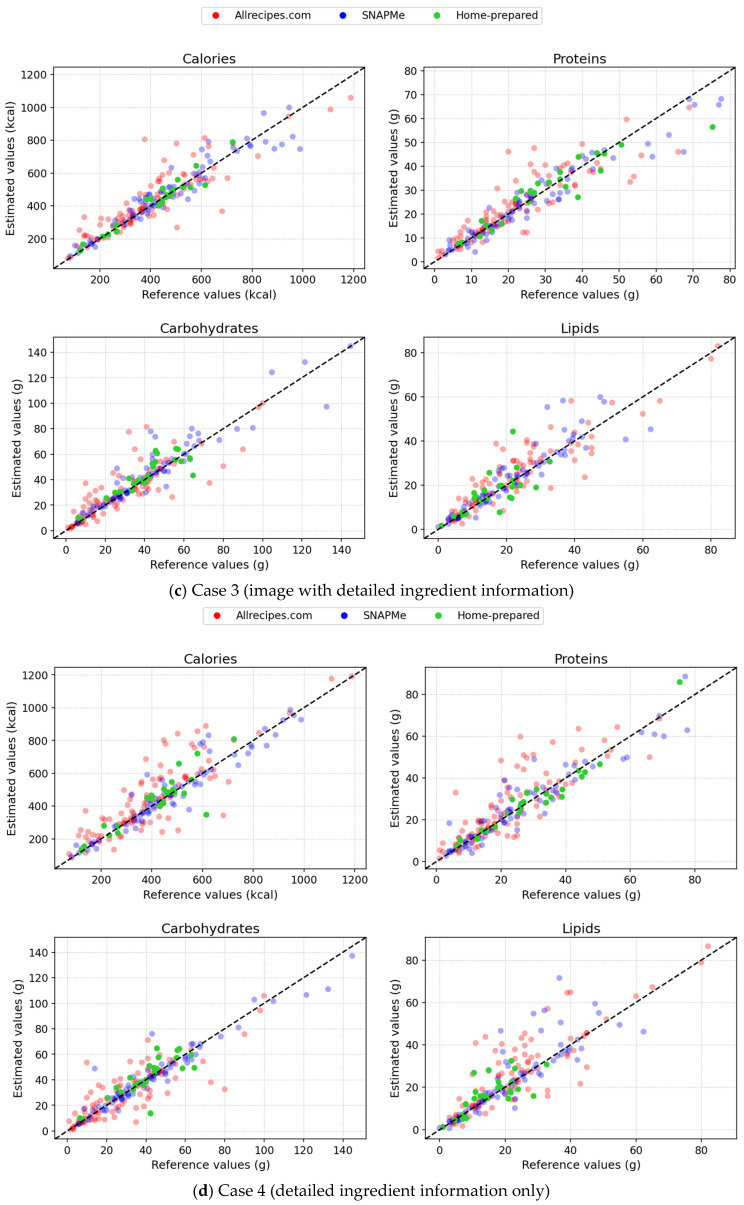
Scatter plots of estimated vs. reference values for calories and macronutrients by data source.

**Figure 2:**
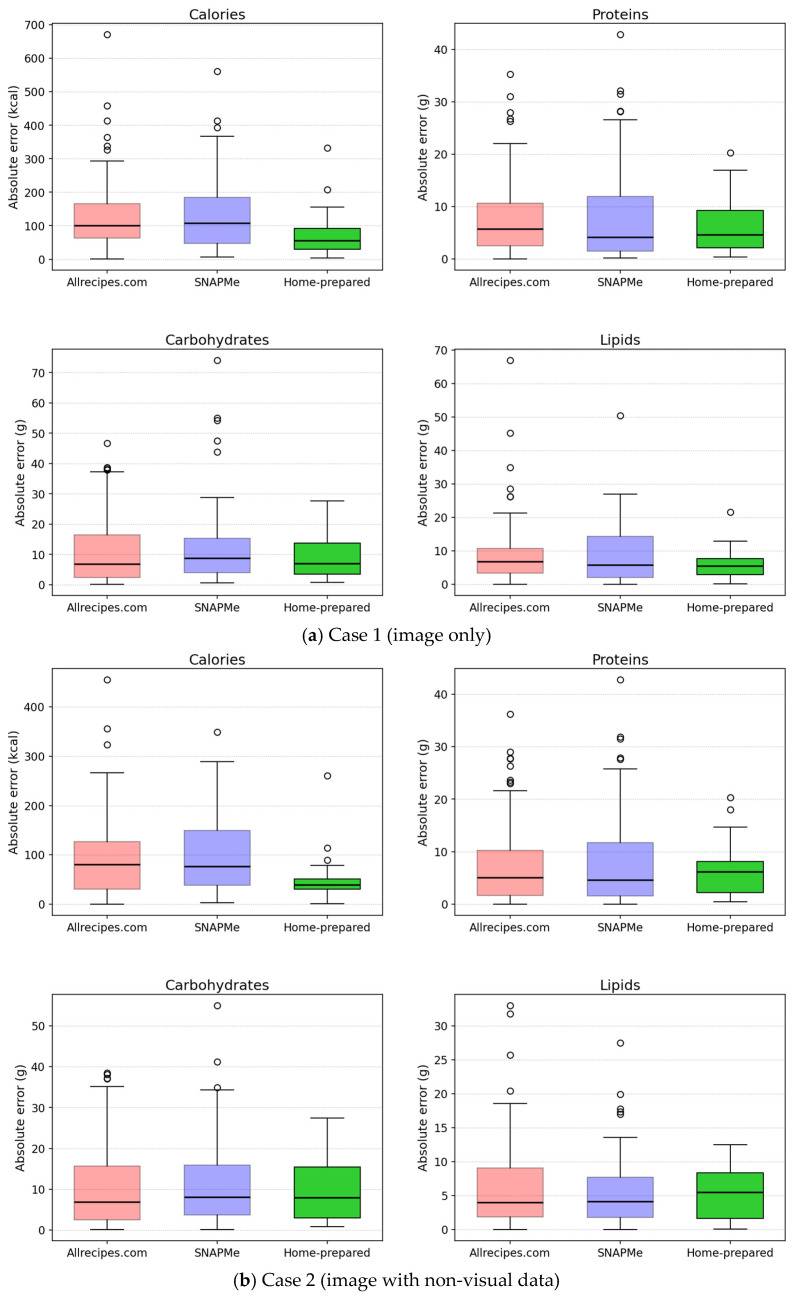

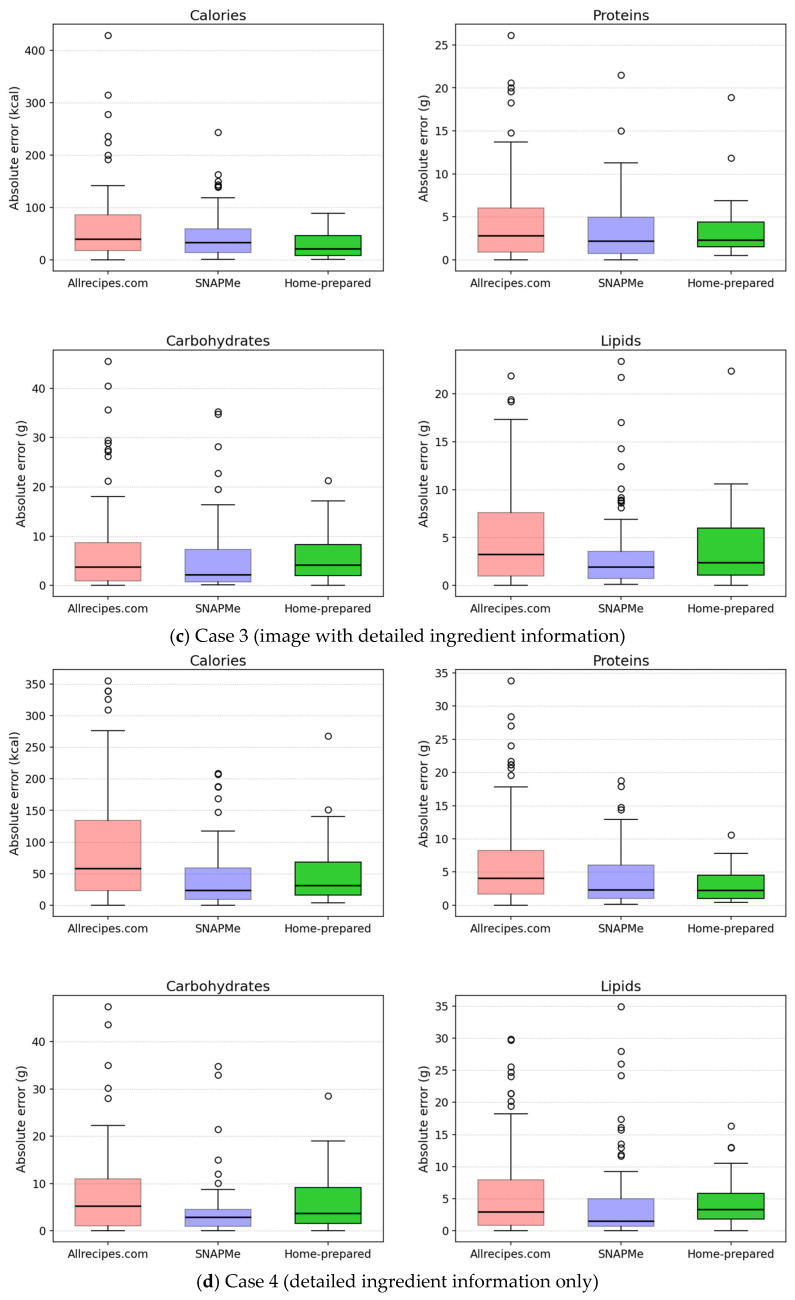
Boxplots of absolute errors for calories and macronutrients by data source. Each boxplot displays the median, IQR, and Tukey whiskers (1.5 × IQR). Data points lying beyond the whiskers are presented as outliers.

All analyses were conducted in Python 3.12, using NumPy 1.26.4, pandas 2.3.3, SciPy 1.16.2, and Matplotlib 3.10.6. Results are grouped by test case and structured around the three reference data sources: Allrecipes.com [20], SNAPMe [11], and Home-prepared. The summary tables (Table 2, Table 3, Table 4 and Table 5) report MAE, MedAE, IQRAE, and RMSE in absolute terms (kcal or g), as well as MAPE, MedAPE, and IQRAPE in percentage (%). The values for each independent data source and for all combined data sources (global) are presented as overall results and by quartile. Scatter plots in Figure 1 then compare estimated calories and macronutrients against the corresponding reference values, while boxplots in Figure 2 complement these evaluations by summarizing the distribution and variability of absolute errors across sources, enabling visual assessment of consistency, dispersion, and the presence of outliers.

**Figure 3:**
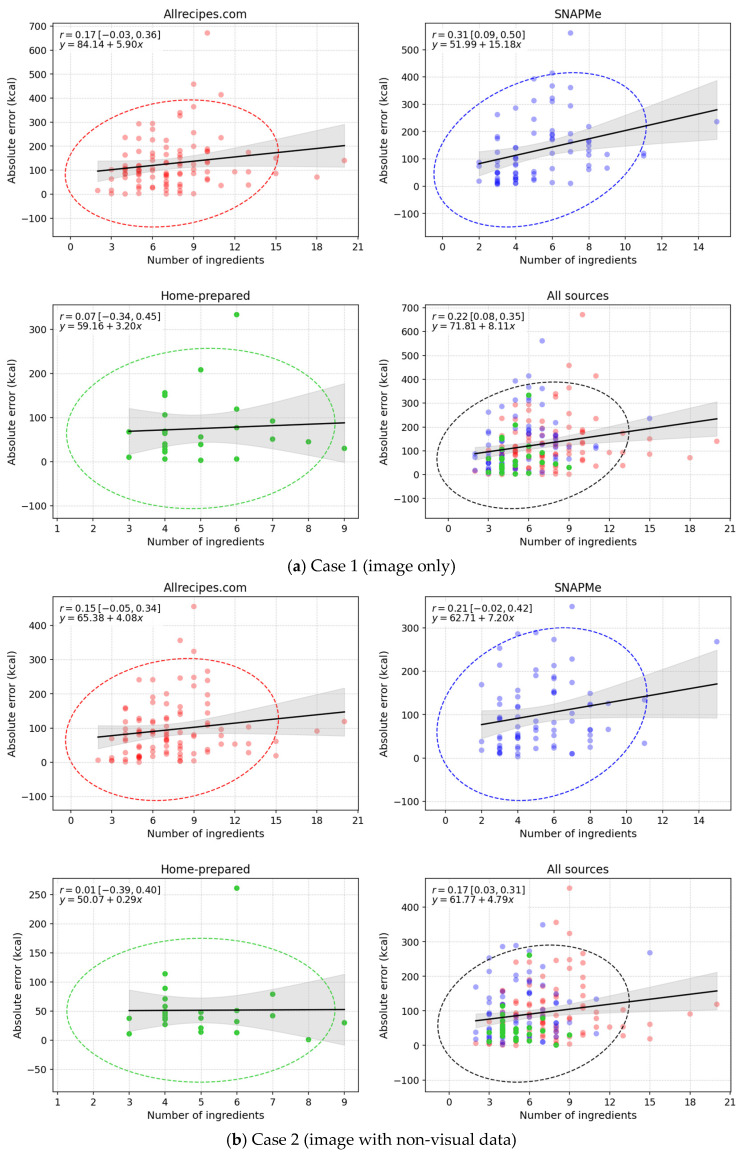

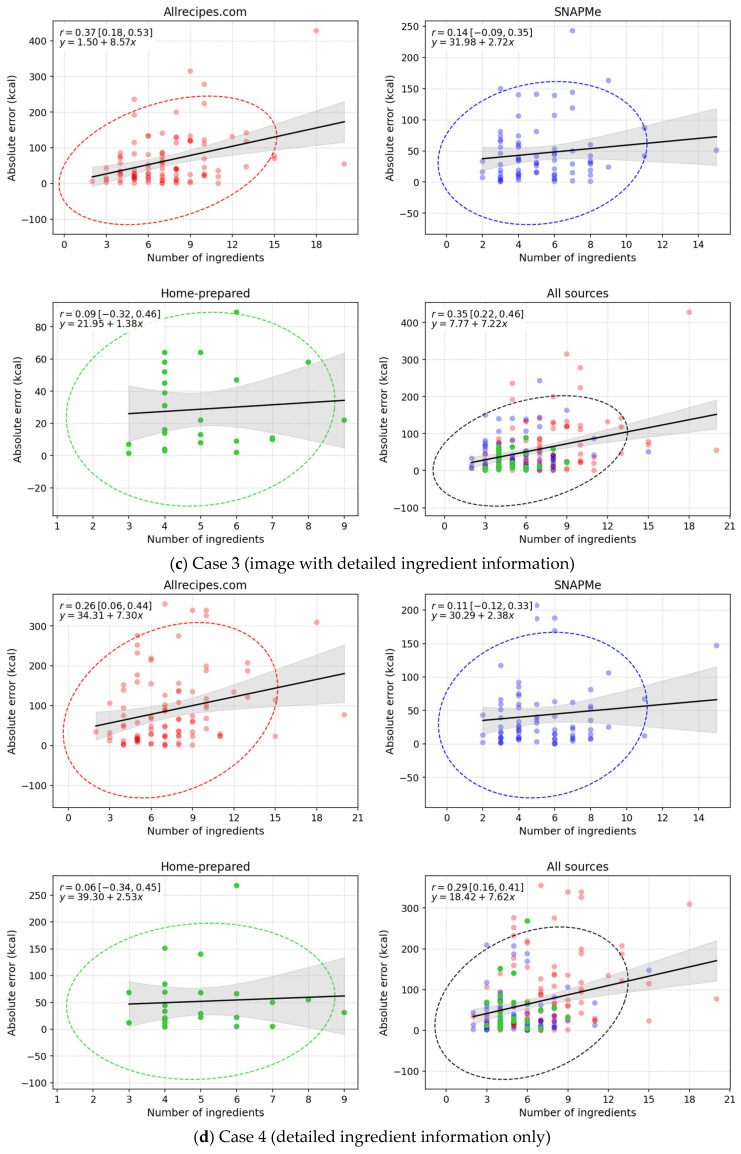
Linear regression analysis between the number of ingredients and the absolute estimation error (kcal) by data source. The plots display Pearson’s correlation coefficient r with its 95% CIs in brackets, along with the fitted linear regression equation. The shaded band represents the 95% CI, and the ellipse illustrates the joint confidence region between predicted and observed values.

The last plots in Figure 3 analyze the relationship between the number of ingredients and the absolute error in energy estimation (kcal). The regression slope indicates the average change in error per additional ingredient, and the Pearson correlation coefficient (r) quantifies the strength of the association. A pronounced positive slope generally corresponds to r values close to 1 (≥0.7), whereas low r values (0–0.3) indicate high variability [9]. The shaded area represents the 95% CI for the regression line, and the ellipse delineates the joint confidence region between predicted and observed values, providing a visual summary of the dispersion and overall fit for each data source.

Finally, nonparametric inferential analyses (Table 6) were performed to assess differences in estimation errors among the three data sources. The Kruskal–Wallis test was applied to each variable across the test cases. When overall differences were significant, pairwise Mann–Whitney tests with Holm correction were conducted. Cliff’s δ was computed to quantify effect sizes. Results are summarized in Table 6, with statistically significant differences ($p_{Holm}$ < 0.05) shown in bold.

**Table 2:** Case 1 (image only). Evaluation metrics for caloric and macronutrient estimation by data source and for all combined data sources.

**Calories (kcal)**								
**Source**	**Range**	**MAE**	**MedAE**	**IQRAE**	**MAPE (%)**	**MedAPE (%)**	**IQRAPE (%)**	**RMSE**
Allrecipes.com	Total	127.81	100.50	102.75	35.00	25.65	27.83	166.99
	95% CIs	107.41, 150.62	87.00, 121.50	72.250, 136.250	29.30, 41.18	23.02, 34.76	19.76, 37.59	135.27, 201.55
	Q1 (≤280)	96.42	85.00	97.00	55.08	50.90	64.61	120.30
	Q2 (280–368.5)	85.13	72.00	89.00	26.53	22.86	26.25	109.46
	Q3 (368.5–502.5)	113.25	96.00	61.50	26.59	23.14	17.84	123.37
	Q4 (>502.5)	216.00	161.50	197.50	30.61	25.65	25.07	264.13
SNAPMe	Total	133.00	108.50	138.25	28.19	28.03	28.93	175.61
	95% CIs	107.73, 160.46	75.00, 135.50	108.49, 189.7	23.26, 33.78	21.00, 30.89	20.68, 32.37	142.51, 208.48
	Q1 (≤348.2)	82.10	73.00	63.50	37.03	29.00	27.75	105.90
	Q2 (348.2–435.5)	72.38	41.50	96.50	18.68	9.84	26.11	95.63
	Q3 (435.5–592.8)	136.90	125.00	91.25	26.46	24.67	15.12	155.84
	Q4 (>592.8)	237.63	245.00	210.00	30.01	32.29	25.56	277.88
Home-prepared	Total	75.14	56.00	62.00	20.12	13.25	13.05	104.52
	95% CIs	49.60, 105.76	34.00, 77.00	30.00, 123.00	12.46, 30.93	10.04, 20.15	7.38, 24.58	63.22, 144.96
	Q1 (≤381)	56.07	40.00	39.25	32.24	20.15	17.85	70.72
	Q2 (381–448)	38.66	39.50	28.25	9.02	8.96	6.24	43.96
	Q3 (448–489)	58.83	57.50	31.75	12.33	11.94	6.76	68.69
	Q4 (>489)	150.16	131.00	99.50	24.87	20.29	14.46	181.76
All sources	Total	123.03	98.00	123.00	30.51	24.34	27.49	163.80
	95% CIs	108.61, 138.78	85.00, 106.00	100.00, 141.00	26.86, 34.25	22.68, 28.49	22.76, 31.71	142.58, 186.00
	Q1 (≤299)	81.69	68.00	89.00	45.21	32.07	52.18	105.20
	Q2 (299–407)	86.20	81.00	93.00	24.44	23.02	24.86	108.5
	Q3 (407–533.5)	98.81	92.50	80.50	21.25	20.73	15.17	113.94
	Q4 (>533.5)	224.91	190.00	208.00	30.94	30.89	26.20	266.83
**Proteins (g)**								
**Source**	**Range**	**MAE**	**MedAE**	**IQRAE**	**MAPE (%)**	**MedAPE (%)**	**IQRAPE (%)**	**RMSE**
Allrecipes.com	Total	8.02	5.70	38.07	51.73	28.40	37.89	11.08
	95% CIs	6.52, 9.60	4.20, 7.60	5.55, 12.90	37.92, 68.77	23.16, 36.36	25.89, 60.04	9.04, 13.02
	Q1 (≤12)	4.51	2.30	3.60	93.82	40.00	90.95	8.06
	Q2 (12–19)	7.72	3.55	8.92	48.02	24.04	57.17	10.83
	Q3 (19–28)	8.66	7.52	12.25	37.05	30.56	23.48	14.43
	Q4 (>28)	11.46	8.00	12.25	25.15	26.00	23.33	14.43
SNAPMe	Total	8.32	4.15	10.54	37.01	21.55	29.65	12.50
	95% CIs	6.30, 10.53	2.80, 6.32	6.10, 14.92	26.20, 51.61	14.67, 32.22	24.33, 36.30	9.60, 15.24
	Q1 (≤13.2)	3.66	1.38	2.60	55.59	22.92	25.61	5.88
	Q2 (13.2–22.6)	6.08	2.60	3.97	37.26	13.61	27.54	10.02
	Q3 (22.6–33.9)	7.30	6.45	7.30	26.13	24.27	27.13	8.76
	Q4 (>33.9)	16.06	14.20	20.25	28.49	31.94	29.88	20.16
Home-prepared	Total	6.14	4.60	7.14	23.93	18.00	18.83	8.04
	95% CIs	4.24, 8.27	2.10, 9.00	3.20, 9.50	14.76, 37.91	8.78, 23.79	8.75, 27.96	5.70, 10.32
	Q1 (≤18.2)	3.07	2.10	2.57	35.52	17.86	19.41	4.52
	Q2 (18.2–27)	5.09	4.85	4.50	20.75	20.19	19.04	6.06
	Q3 (27–38.8)	4.04	3.35	2.87	11.61	10.51	8.98	4.87
	Q4 (>38.8)	12.86	11.45	6.80	25.90	24.50	13.11	13.61
All sources	Total	7.89	5.00	8.50	42.58	24.84	30.97	11.32
	95% CIs	6.81, 9.09	4.20, 6.30	6.65, 11.20	4.17, 52.43	20.38, 28.53	26.08, 37.07	9.77, 12.89
	Q1 (≤13)	3.98	2.20	3.30	70.10	27.27	86.67	6.81
	Q2 (13–22)	7.53	4.60	8.01	43.69	24.29	52.32	10.63
	Q3 (22–33.3)	7.12	7.20	5.40	26.59	24.84	22.75	8.64
	Q4 (>33.3)	13.16	9.70	15.00	25.69	23.79	26.92	16.69
**Carbohydrates (g)**								
**Source**	**Range**	**MAE**	**MedAE**	**IQRAE**	**MAPE (%)**	**MedAPE (%)**	**IQRAPE (%)**	**RMSE**
Allrecipes.com	Total	11.48	6.80	14.12	53.87	37.08	51.06	16.34
	95% CIs	9.27, 13.85	5.05, 10.45	9.07, 19.12	41.87, 67.89	30.00, 45.00	36.28, 67.94	13.47, 18.99
	Q1 (≤12)	6.13	2.80	6.75	90.43	48.47	66.93	9.97
	Q2 (12–26)	10.14	7.40	9.00	51.92	39.47	48.26	13.80
	Q3 (26–41)	15.42	12.15	19.87	45.07	30.00	62.85	19.96
	Q4 (>41)	14.75	12.00	15.10	23.67	22.22	31.35	19.91
SNAPMe	Total	12.99	8.75	11.50	32.01	24.41	26.79	18.86
	95% CIs	10.07, 16.33	5.98, 11.50	8.10, 18.76	26.09, 38.62	18.57, 33.70	21.45, 37.98	13.74, 23.86
	Q1 (≤26)	7.98	4.32	5.25	48.08	36.65	60.32	11.05
	Q2 (26–40)	7.12	4.80	8.60	22.68	14.08	26.10	8.59
	Q3 (40–52.3)	13.97	11.75	8.77	30.13	25.57	17.52	17.43
	Q4 (>52.3)	22.60	12.50	18.65	25.29	19.78	22.56	30.06
Home-prepared	Total	8.91	7.10	10.31	25.59	17.13	21.95	11.56
	95% CIs	6.12, 11.95	3.76, 12.50	5.90, 16.20	16.22, 37.54	11.36, 30.29	11.19, 41.17	8.23, 14.58
	Q1 (≤33.5)	11.71	14.50	13.75	49.17	48.44	36.84	14.87
	Q2 (33.5–41.8)	7.20	7.12	4.13	18.57	17.44	12.34	8.07
	Q3 (41.8–46.4)	6.30	4.90	8.75	14.22	10.85	20.90	8.12
	Q4 (>550)	9.97	6.68	14.21	16.47	10.81	21.74	12.95
All sources	Total	11.72	8.10	12.10	41.95	29.55	37.11	16.84
	95% CIs	10.11, 13.50	6.00, 10.20	9.60, 16.05	35.31, 49.61	23.08, 32.59	30.52, 45.87	14.36, 19.43
	Q1 (≤17.9)	6.01	3.40	6.50	68.97	45.00	55.13	9.24
	Q2 (17.9–34.5)	11.46	7.70	12.00	45.50	36.33	42.82	15.25
	Q3 (34.5–46.2)	11.33	9.65	11.05	28.14	25.10	25.24	14.55
	Q4 (>46.2)	18.08	12.00	20.00	24.90	19.78	25.32	24.56
**Lipids (g)**								
**Source**	**Range**	**MAE**	**MedAE**	**IQRAE**	**MAPE (%)**	**MedAPE (%)**	**IQRAPE (%)**	**RMSE**
Allrecipes.com	Total	9.17	6.80	7.45	59.76	37.71	38.18	13.35
	95% CIs	7.39, 11.24	5.30, 8.50	6.00, 11.52	43.76, 80.22	28.42, 47.00	30.90, 61.71	9.72, 17.26
	Q1 (≤10)	6.33	4.50	6.50	128.30	89.00	91.25	8.49
	Q2 (10–19.5)	5.81	5.40	6.65	40.56	41.11	32.42	7.08
	Q3(19.5–27.2)	6.54	5.65	6.27	27.14	22.09	25.75	8.12
	Q4 (>27.2)	17.95	15.05	12.12	39.36	37.74	31.88	22.89
SNAPMe	Total	8.90	5.79	12.32	46.82	41.33	39.29	12.55
	95% CIs	7.00, 11.01	4.30, 8.30	7.60, 14.75	38.03, 57.26	35.62, 49.09	24.02, 55.37	9.76, 15.60
	Q1 (≤11.2)	3.52	2.60	3.50	67.91	53.96	29.06	4.79
	Q2 (11.2–19.2)	6.49	5.14	4.70	44.70	35.97	33.94	8.43
	Q3 (19.2–30.8)	8.13	7.31	11.65	34.11	33.05	57.71	10.72
	Q4 (>30.8)	17.29	16.60	9.90	40.89	41.39	19.04	20.35
Home-prepared	Total	6.36	5.52	4.80	44.20	35.20	27.75	7.98
	95% CIs	4.63, 8.32	3.10, 7.40	2.10, 9.91	31.81, 60.03	27.57, 42.78	9.10, 47.95	5.67, 10.34
	Q1 (≤10.5)	4.32	2.90	3.80	66.29	30.00	71.45	5.75
	Q2 (10.5–15)	4.05	4.80	2.30	31.38	35.93	15.29	4.47
	Q3 (15–21.7)	7.56	6.95	1.67	38.13	35.11	11.91	7.97
	Q4 (>21.7)	9.84	10.05	8.32	37.34	35.23	34.61	11.96
All sources	Total	8.70	6.00	9.10	52.88	39.48	38.53	12.48
	95% CIs	7.51, 10.05	5.24, 7.40	7.00, 11.50	43.89, 63.64	34.10, 43.75	31.87, 47.86	10.27, 14.94
	Q1 (≤10.6)	4.82	3.40	4.70	98.28	61.17	88.54	6.78
	Q2 (10.6–18.7)	6.09	5.24	5.10	43.46	36.67	32.92	7.53
	Q3(18.7–27.5)	6.65	5.88	7.30	29.32	28.21	32.83	8.27
	Q4 (>27.5)	17.21	15.40	13.10	40.92	41.13	30.58	21.21

**Note:** Values for each independent data source and for all combined data sources (global) are presented as overall results and by quartile. 95% CIs for all reported metrics were estimated via nonparametric bootstrap (10,000 resamples, dish-level).

**Table 3:** Case 2 (image with non-visual data). Evaluation metrics for caloric and macronutrient estimation by data source and for all combined data sources.

**Calories (kcal)**								
**Source**	**Range**	**MAE**	**MedAE**	**IQRAE**	**MAPE (%)**	**MedAPE (%)**	**IQRAPE (%)**	**RMSE**
Allrecipes.com	Total	95.58	80.15	96.50	28.85	18.95	32.82	127.45
	95% CIs	79.33, 113.22	62.00, 91.00	71.50, 128.75	23.40, 34.71	16.11, 24.30	19.54, 43.54	104.25, 150.58
	Q1 (≤280)	88.85	81.00	81.00	50.13	43.03	52.36	107.63
	Q2 (280–368.5)	70.13	43.00	68.50	21.66	13.27	21.88	100.38
	Q3 (368.5–502.5)	97.79	75.50	68.75	23.27	17.60	14.29	135.15
	Q4 (>502.5)	124.79	99.50	112.75	19.15	16.95	17.68	158.06
SNAPMe	Total	101.13	77.00	111.25	22.17	19.32	21.53	129.39
	95% CIs	82.99, 119.88	54.50, 110.00	81.00, 143.75	18.63, 25.94	12.88, 25.65	17.41, 25.78	107.67, 149.83
	Q1 (≤348.2)	78.21	64.00	78.50	33.05	28.49	26.28	102.59
	Q2 (348.2–435.5)	49.22	39.50	33.00	12.73	10.50	7.89	60.87
	Q3 (435.5–592.8)	110.40	125.00	97.00	21.31	25.58	18.56	130.05
	Q4 (>592.8)	164.47	156.00	142.50	21.03	19.06	18.96	187.47
Home-prepared	Total	51.50	39.00	21.00	13.55	10.41	9.00	71.37
	95% CIs	35.74, 73.72	32.00, 49.00	11.50, 57.00	9.26, 19.02	7.69, 12.78	4.02, 15.98	41.12, 104.46
	Q1 (≤381)	38.07	37.50	16.50	20.08	17.67	7.66	41.84
	Q2 (381–448)	44.16	42.50	31.00	10.48	10.16	8.26	56.99
	Q3 (448–489)	36.50	34.00	13.75	7.69	7.18	3.06	42.06
	Q4 (>489)	89.50	53.00	37.7	14.84	9.34	3.79	119.02
All sources	Total	92.04	68.00	95.50	24.35	17.67	23.36	122.48
	95% CIs	80.99, 103.63	54.00, 83.00	79.50, 118.50	21.21, 27.67	14.36, 20.15	19.01, 28.18	107.89, 137.76
	Q1 (≤299)	68.03	53.00	61.00	37.40	28.49	46.45	87.27
	Q2 (299–407)	83.32	53.00	80.00	23.69	13.74	26.45	119.37
	Q3 (407–533.5)	73.79	64.50	78.25	15.81	14.06	15.89	90.77
	Q4 (>533.5)	142.63	119.00	149.00	20.33	17.57	21.54	172.54
**Proteins (g)**								
**Source**	**Range**	**MAE**	**MedAE**	**IQRAE**	**MAPE (%)**	**MedAPE (%)**	**IQRAPE (%)**	**RMSE**
Allrecipes.com	Total	7.47	5.10	8.52	47.66	27.30	40.52	10.77
	95% CIs	5.96, 9.11	3.50, 6.30	5.57, 11.72	34.97, 63.14	22.38, 35.71	29.99, 55.12	8.62, 12.80
	Q1 (≤12)	3.43	2.30	2.20	81.23	23.00	63.79	6.48
	Q2 (12–19)	7.49	5.20	9.60	47.02	34.73	59.71	10.67
	Q3 (19–28)	8.58	7.60	7.45	36.79	30.70	29.98	10.55
	Q4 (>28)	10.67	6.10	9.70	23.19	19.68	20.95	14.27
SNAPMe	Total	8.11	4.62	10.09	36.11	24.55	29.51	12.06
	95% CIs	6.16, 10.24	3.10, 6.77	6.22, 13.92	25.84, 50.28	17.23, 32.57	23.42, 37.84	9.17, 14.85
	Q1 (≤13.2)	3.95	2.40	2.93	59.61	27.50	45.65	6.02
	Q2 (13.2–22.6)	4.76	2.65	4.12	29.10	16.28	25.61	7.61
	Q3 (22.6–33.9)	7.61	7.95	6.96	26.97	28.21	26.18	9.01
	Q4 (>33.9)	15.93	15.30	20.70	27.93	31.61	28.47	19.97
Home-prepared	Total	6.55	6.20	5.90	24.27	18.24	12.88	8.29
	95% CIs	4.70, 8.63	4.40, 7.90	2.56, 8.27	16.82, 34.05	15.59, 26.96	5.92, 29.56	5.93, 10.55
	Q1 (≤18.2)	3.73	2.20	5.82	35.19	15.71	34.88	4.79
	Q2 (18.2–27)	4.05	4.85	4.42	16.58	20.19	18.10	4.91
	Q3 (27–38.8)	5.90	5.75	2.92	17.23	16.91	7.94	6.48
	Q4 (>38.8)	13.00	11.85	8.85	26.25	22.48	17.04	13.91
All sources	Total	7.59	5.10	8.35	40.28	25.53	32.99	11.01
	95% CIs	6.52, 8.76	4.30, 6.10	6.55, 11.05	32.54, 49.35	21.43, 28.57	26.65, 39.83	9.45, 12.57
	Q1 (≤13)	3.61	2.30	3.00	65.31	27.27	49.83	5.99
	Q2 (13–22)	7.07	5.00	6.67	40.88	27.39	38.63	9.94
	Q3 (22–33.3)	6.76	6.10	7.50	25.18	25.53	28.30	8.49
	Q4 (>33.3)	13.17	9.30	13.70	25.85	24.64	25.80	16.71
**Carbohydrates (g)**								
**Source**	**Range**	**MAE**	**MedAE**	**IQRAE**	**MAPE (%)**	**MedAPE (%)**	**IQRAPE (%)**	**RMSE**
Allrecipes.com	Total	10.76	6.90	13.20	50.86	32.41	47.70	15.02
	95% CIs	8.78, 12.89	4.95, 10.60	9.92, 17.30	39.51, 64.05	22.50, 40.71	35.42, 73.78	12.56, 17.37
	Q1 (≤12)	5.98	2.70	6.15	84.98	42.94	89.27	10.14
	Q2 (12–26)	9.41	7.00	10.05	49.64	35.79	46.10	12.98
	Q3 (26–41)	14.64	12.95	14.57	42.77	33.68	45.60	18.54
	Q4 (>41)	13.46	11.40	16.00	21.94	22.22	24.53	17.29
SNAPMe	Total	11.29	8.05	12.27	27.74	23.26	24.64	15.37
	95% CIs	9.05, 13.78	5.37, 11.15	8.36, 17.21	23.05, 33.00	18.41, 27.80	17.16, 32.91	12.13, 18.77
	Q1 (≤26)	6.68	4.15	4.85	38.50	24.05	46.35	9.61
	Q2 (26–40)	7.67	5.70	9.00	24.01	17.07	24.98	9.12
	Q3 (40–52.3)	11.77	10.40	9.52	25.27	22.37	19.26	13.91
	Q4 (>52.3)	18.90	14.20	18.50	22.10	24.64	16.05	23.76
Home-prepared	Total	9.50	8.00	12.50	26.80	17.67	27.64	12.19
	95% CIs	6.56, 12.58	3.00, 13.30	5.75, 18.00	17.39, 38.87	11.25, 33.33	13.21, 46.38	8.86, 15.14
	Q1 (≤33.5)	11.63	13.30	14.30	48.39	48.44	36.08	14.82
	Q2 (33.5–41.8)	8.70	7.57	4.41	22.43	18.56	13.09	10.61
	Q3 (41.8–46.4)	7.13	5.60	8.05	15.99	12.25	19.05	8.91
	Q4 (>550)	10.17	6.68	15.26	16.81	10.81	23.60	13.08
All sources	Total	10.80	7.50	12.65	39.00	24.81	36.24	14.83
	95% CIs	9.41, 12.26	6.00, 9.40	10.35, 15.85	32.90, 46.03	21.42, 30.00	27.11, 42.19	12.99, 16.67
	Q1 (≤17.9)	5.55	3.20	6.10	63.19	38.33	59.76	8.78
	Q2 (17.9–34.5)	10.79	6.80	11.80	42.69	34.78	41.52	14.50
	Q3 (34.5–46.2)	11.06	9.45	11.78	27.58	23.57	25.78	13.89
	Q4 (>46.2)	15.79	12.20	17.70	22.31	22.22	20.86	19.96
**Lipids (g)**								
**Source**	**Range**	**MAE**	**MedAE**	**IQRAE**	**MAPE (%)**	**MedAPE (%)**	**IQRAPE (%)**	**RMSE**
Allrecipes.com	Total	6.25	4.00	7.16	51.17	22.08	39.88	9.06
	95% CIs	4.97, 7.60	2.70, 5.25	4.37, 9.82	31.32, 79.60	15.45, 30.77	27.46, 60.82	7.11, 10.96
	Q1 (≤10)	5.81	3.30	7.10	121.44	62.50	68.33	9.63
	Q2 (10–19.5)	5.54	2.70	4.30	37.66	24.55	39.95	7.93
	Q3 (19.5–27.2)	4.31	2.60	3.55	17.65	9.92	14.51	6.16
	Q4 (>27.2)	9.32	8.70	10.2	24.44	20.88	22.67	11.55
SNAPMe	Total	5.61	4.19	5.90	35.15	22.73	39.83	7.76
	95% CIs	4.44, 6.88	3.00, 4.95	3.40, 9.20	27.06, 44.78	18.92, 31.80	22.05, 51.36	6.07, 9.45
	Q1 (≤11.2)	3.08	1.80	3.40	60.51	55.00	51.23	4.31
	Q2 (11.2–19.2)	5.54	4.64	4.37	37.41	32.02	38.52	7.36
	Q3 (19.2–30.8)	5.22	4.20	3.85	22.46	18.10	21.34	7.12
	Q4 (>30.8)	8.57	9.35	8.35	21.01	21.57	22.59	10.80
Home-prepared	Total	5.56	5.52	6.72	36.12	38.25	34.02	6.82
	95% CIs	4.05, 7.10	2.50, 7.90	3.50, 9.10	27.44, 45.07	20.00, 43.91	16.17, 50.45	5.36, 8.11
	Q1 (≤10.5)	3.08	1.68	4.71	41.06	36.36	53.97	4.18
	Q2 (10.5–15)	4.96	4.80	4.12	39.44	39.69	43.04	5.85
	Q3 (15–21.7)	6.47	7.21	2.93	31.94	36.79	12.85	7.35
	Q4 (>21.7)	8.16	9.44	6.55	31.23	39.15	19.99	9.22
All sources	Total	5.92	4.30	6.65	43.20	24.36	40.84	8.32
	95% CIs	5.13, 6.76	3.30, 4.90	5.48, 8.06	32.63, 57.51	20.25, 30.48	31.54, 51.91	7.12, 9.54
	Q1 (≤10.6)	4.21	2.20	4.40	88.29	51.67	70.52	7.29
	Q2 (10.6–18.7)	5.45	4.50	5.20	38.87	29.38	43.65	7.19
	Q3 (18.7–27.5)	5.11	4.25	5.54	22.70	17.62	25.75	6.80
	Q4 (>27.5)	8.90	9.30	9.51	23.45	21.50	23.97	11.19

**Note:** Values for each independent data source and for all combined data sources (global) are presented as overall results and by quartile. 95% CIs for all reported metrics were estimated via nonparametric bootstrap (10,000 resamples, dish-level).

**Table 4:** Case 3 (image with detailed ingredient information). Evaluation metrics for caloric and macronutrient estimation by data source and for all combined data sources.

**Calories (kcal)**								
**Source**	**Range**	**MAE**	**MedAE**	**IQRAE**	**MAPE (%)**	**MedAPE (%)**	**IQRAPE (%)**	**RMSE**
Allrecipes.com	Total	64.99	40.00	68.50	18.64	9.84	20.20	97.84
	95% CIs	51.05, 80.08	26.50, 54.00	52.50, 103.75	14.16, 23.60	8.11, 12.29	11.66, 26.80	73.95, 121.58
	Q1 (≤280)	49.06	38.00	66.00	29.26	14.29	35.63	67.87
	Q2 (280–368.5)	28.50	20.00	19.50	8.87	6.00	6.04	41.19
	Q3 (368.5–502.5)	94.67	63.50	93.50	22.21	15.71	21.32	135.19
	Q4 (>502.5)	86.89	60.00	103.75	13.38	10.45	10.64	116.58
SNAPMe	Total	46.48	33.50	45.75	10.16	8.07	11.64	65.82
	95% CIs	36.38, 57.68	24.00, 42.00	32.20, 65.75	8.13, 12.43	5.12, 10.95	8.58, 14.69	50.56, 80.75
	Q1 (≤348.2)	24.42	15.00	23.50	11.97	8.16	13.50	35.27
	Q2 (348.2–435.5)	36.05	35.00	56.75	9.45	8.78	15.58	45.92
	Q3 (435.5–592.8)	44.45	38.00	36.25	8.82	8.15	6.83	57.15
	Q 4 (>592.8)	80.36	53.00	109.00	10.28	7.39	11.13	102.66
Home-prepared	Total	28.82	22.00	38.00	6.92	6.02	8.17	37.55
	95% CIs	19.88, 38.44	10.00, 45.00	18.00, 51.00	4.84, 9.19	2.44, 9.73	5.09, 11.11	27.21, 46.86
	Q1 (≤281)	19.78	16.00	21.50	8.40	6.02	9.03	27.05
	Q2 (281–448)	35.16	35.00	20.75	8.29	8.45	5.77	38.15
	Q3 (448–489)	7.66	8.50	4.75	1.61	1.79	0.97	8.50
	Q4 (>489)	54.16	58.00	17.25	9.13	9.55	2.57	59.10
All sources	Total	53.33	34.00	56.00	13.92	8.40	12.73	80.86
	95% CIs	45.08, 62.27	27.00, 42.00	44.00, 70.00	11.54, 16.66	7.18, 9.91	10.09, 15.76	65.90, 96.38
	Q1 (≤299)	34.20	24.00	39.00	19.97	9.16	19.11	51.40
	Q2 (299–407)	46.13	29.00	44.00	12.73	8.63	11.57	78.80
	Q3 (407–533.5)	55.58	36.00	46.75	11.96	8.14	10.10	83.77
	Q4 (>533.5)	77.45	56.00	93.00	10.98	8.84	10.48	101.44
**Proteins (g)**								
**Source**	**Range**	**MAE**	**MedAE**	**IQRAE**	**MAPE (%)**	**MedAPE (%)**	**IQRAPE (%)**	**RMSE**
Allrecipes.com	Total	4.59	2.80	5.15	27.24	14.69	27.08	6.91
	95% CIs	3.61, 5.68	2.20, 3.85	3.97, 6.37	19.96, 35.91	12.76, 18.71	16.52, 37.79	5.32, 8.42
	Q1 (≤12)	2.08	1.30	2.70	46.22	21.67	55.83	2.96
	Q2 (12–19)	2.80	2.30	3.15	17.82	12.98	23.00	4.08
	Q3 (19–28)	6.34	3.30	9.95	26.72	14.95	37.02	9.28
	Q4 (>28)	7.37	5.70	3.75	16.99	15.35	10.58	9.07
SNAPMe	Total	3.38	2.20	4.22	15.19	10.17	13.84	5.02
	95% CIs	2.59, 4.27	1.47, 3.25	2.81, 5.50	11.37, 19.93	7.78, 14.68	10.46, 19.33	3.67, 6.39
	Q1 (≤13.2)	1.66	0.96	1.98	24.33	11.49	24.28	2.47
	Q2 (13.2–22.6)	2.26	1.52	3.32	13.21	9.46	16.91	3.19
	Q3 (22.6–33.9)	3.34	2.75	3.70	11.61	10.78	11.87	4.11
	Q4 (>33.9)	6.19	4.70	6.92	11.30	10.33	9.86	8.14
Home-prepared	Total	3.77	2.30	2.90	13.10	12.56	7.97	5.47
	95% CIs	2.45, 5.52	1.60, 4.34	1.70, 5.10	9.90, 16.57	7.86, 15.33	4.77, 18.06	2.98, 7.97
	Q1 (≤18.2)	1.91	1.70	1.24	15.42	13.82	4.09	2.23
	Q2 (18.2–27)	3.23	3.20	2.12	13.30	12.27	10.70	3.68
	Q3 (27–38.8)	4.50	3.80	2.02	13.06	10.92	7.61	5.74
	Q4 (38.8)	5.76	3.25	4.87	10.22	7.99	11.42	8.50
All sources	Total	4.03	2.50	4.30	20.85	12.80	17.03	6.07
	95% CIs	3.41, 4.69	2.20, 3.30	3.75, 5.35	16.91, 25.71	10.33, 14.71	13.55, 23.12	5.02, 7.09
	Q1 (≤13)	1.85	0.96	2.27	32.85	13.82	35.00	2.68
	Q2 (13–22)	3.20	2.29	2.47	18.17	12.61	13.61	5.32
	Q3 (22–33.3)	4.68	3.60	4.85	17.46	14.23	16.42	6.38
	Q4 (>33.3)	6.66	5.20	5.80	13.59	12.79	10.67	8.62
**Carbohydrates (g)**								
**Source**	**Range**	**MAE**	**MedAE**	**IQRAE**	**MAPE (%)**	**MedAPE (%)**	**IQRAPE (%)**	**RMSE**
Allrecipes.com	Total	6.89	3.80	7.70	33.52	15.65	34.01	11.55
	95% CIs	5.15, 8.83	2.20, 4.70	4.37, 12.82	24.60, 44.09	10.93, 22.86	23.02, 54.35	8.58, 14.34
	Q1 (≤12)	4.64	1.30	4.60	60.21	28.57	66.04	7.93
	Q2 (12–26)	5.68	3.90	6.05	31.15	22.94	30.77	7.85
	Q3 (26–41)	8.85	4.15	7.25	25.34	11.07	19.11	15.12
	Q4 (>41)	8.61	3.50	10.65	14.26	8.33	21.75	13.63
SNAPMe	Total	5.51	2.20	6.62	13.41	6.13	12.08	9.42
	95% CIs	3.89, 7.37	1.20, 3.00	3.20, 10.34	9.63, 17.82	5.00, 8.93	6.99, 21.89	6.58, 12.11
	Q1 (≤26)	3.13	1.00	2.42	17.12	6.11	12.67	6.09
	Q2 (26–40)	2.54	1.60	1.50	8.24	5.33	5.70	3.93
	Q3 (40–52.3)	8.05	3.05	11.00	17.94	6.13	25.51	12.70
	Q4 (>52.3)	8.29	6.90	9.36	9.84	8.92	11.46	11.81
Home-prepared	Total	5.56	4.20	6.30	15.04	10.95	13.60	7.67
	95% CIs	3.62, 7.77	2.20, 6.80	3.01, 9.19	10.17, 20.60	6.57, 19.55	7.06, 25.85	4.86, 10.24
	Q1 (≤33.5)	4.05	3.60	2.35	22.09	19.60	15.60	4.71
	Q2 (33.5–41.8)	1.44	1.67	1.81	3.82	4.52	4.77	1.75
	Q3 (41.8–46.4)	9.18	8.55	6.32	20.26	19.11	13.37	10.60
	Q4 (>46.4)	7.80	6.50	3.22	12.80	10.89	6.26	10.20
All sources	Total	6.20	2.90	7.36	23.52	10.40	22.58	10.34
	95% CIs	5.12, 7.41	2.20, 4.00	5.05, 8.75	18.61, 29.20	8.28, 12.92	17.84, 28.84	8.47, 12.20
	Q1 (≤17.9)	4.16	2.20	4.50	44.80	22.94	48.72	6.79
	Q2 (17.9–34.5)	5.23	2.20	4.30	19.83	6.57	17.27	9.42
	Q3 (34.5–46.2)	6.76	2.75	7.67	16.48	7.13	18.23	11.46
	Q4 (>46.2)	8.66	6.20	12.70	12.82	9.17	14.58	12.73
**Lipids (g)**								
**Source**	**Range**	**MAE**	**MedAE**	**IQRAE**	**MAPE (%)**	**MedAPE (%)**	**IQRAPE (%)**	**RMSE**
Allrecipes.com	Total	4.81	3.25	6.62	27.20	16.34	32.92	6.82
	CI95%	3.87, 5.80	2.30, 4.30	4.50, 7.72	21.55, 33.27	10.42, 23.33	21.46, 42.80	5.58, 8.03
	Q1 (≤10)	2.23	0.70	2.80	35.15	17.14	57.00	3.73
	Q2 (10–19.5)	5.67	4.50	7.13	36.99	28.12	45.46	7.89
	Q3 (19.5–27.2)	4.69	3.83	4.57	19.27	15.82	18.50	6.02
	Q4 (>27.2)	6.78	5.35	7.15	17.46	10.12	19.39	8.69
SNAPMe	Total	3.54	1.92	2.81	18.96	12.44	21.28	5.85
	95% CIs	2.57, 4.67	1.40, 2.60	1.80, 5.65	14.89, 23.47	8.87, 16.86	12.31, 31.03	3.99, 7.59
	Q1 (≤11.2)	1.54	1.10	1.85	30.11	23.61	41.33	2.08
	Q2 (11.2–19.2)	2.67	1.85	1.35	17.02	13.36	10.21	3.66
	Q3 (19.2–30.8)	2.67	1.50	2.45	11.37	6.59	11.48	3.83
	Q4 (>30.8)	7.19	3.60	9.00	17.43	9.68	19.40	10.12
Home-prepared	Total	4.26	2.40	4.92	27.64	21.74	26.94	6.48
	95% CIs	2.54, 6.38	1.20, 5.00	2.60, 9.10	18.35, 37.86	9.44, 33.80	17.43, 51.00	3.63, 9.33
	Q1 (≤10.5)	1.57	1.07	0.69	26.55	15.00	32.28	2.42
	Q2 (10.5–15)	3.78	3.60	2.95	28.93	31.59	24.08	5.10
	Q3 (15–21.7)	4.95	4.45	5.32	25.13	21.01	23.77	5.97
	Q4 (>21.7)	7.19	4.55	5.99	30.14	19.41	21.90	10.33
All sources	Total	4.26	2.50	5.02	24.16	14.94	27.87	6.42
	95% CIs	3.62, 4.97	2.00, 3.00	3.64, 6.85	20.67, 27.90	11.43, 17.78	21.39, 34.87	5.45, 7.38
	Q1 (≤10.6)	1.77	0.70	2.10	31.69	16.90	49.87	2.94
	Q2 (10.6–18.7)	3.99	2.50	4.57	27.35	17.69	29.44	5.67
	Q3 (18.7–27.5)	4.57	3.40	4.62	20.31	13.81	20.22	6.55
	Q4 (>27.5)	6.71	4.30	7.69	17.36	9.77	20.17	9.02

**Note:** Values for each independent data source and for all combined data sources (global) are presented as overall results and by quartile. 95% CIs for all reported metrics were estimated via nonparametric bootstrap (10,000 resamples, dish-level).

**Table 5:** Case 4 (detailed ingredient information only). Evaluation metrics for caloric and macronutrient estimation by data source and for all combined data sources.

**Calories (kcal)**								
**Source**	**Range**	**MAE**	**MedAE**	**IQRAE**	**MAPE (%)**	**MedAPE (%)**	**IQRAPE (%)**	**RMSE**
Allrecipes.com	Total	88.42	58.00	111.25	25.76	17.24	34.61	125.75
	95% CIs	70.69, 106.54	35.00, 77.00	76.75, 136.75	20.37, 31.41	8.97, 23.86	24.81, 42.84	102.82, 147.11
	Q1 (≤280)	61.60	57.00	73.00	37.19	23.84	39.21	81.64
	Q2 (280–368.5)	61.06	25.00	94.50	18.54	7.82	28.58	89.71
	Q3 (368.5–502.5)	127.52	104.00	137.80	29.51	25.73	32.89	167.50
	Q4 (>502.5)	103.46	55.00	137.50	17.02	9.12	22.69	143.33
SNAPMe	Total	43.01	23.50	50.00	10.13	5.80	11.24	66.11
	95% CIs	32.15, 55.00	16.00, 35.50	31.00, 62.50	7.56, 13.06	4.35, 8.46	7.29, 14.74	48.67, 81.84
	Q1 (≤348.2)	28.89	16.00	30.50	13.35	8.46	11.79	44.82
	Q2 (348.2–435.5)	33.78	19.50	30.00	8.76	5.04	7.57	57.57
	Q3 (435.5–592.8)	54.51	33.50	60.25	10.56	6.52	13.27	76.65
	Q4 (>592.8)	55.00	36.00	60.00	7.80	4.33	6.77	79.40
Home-prepared	Total	51.95	31.00	52.00	11.91	9.47	11.39	78.09
	95% CIs	31.90, 76.38	19.00, 66.00	23.00, 73.00	8.11, 16.25	4.75, 14.20	6.69, 20.07	42.22, 112.64
	Q1 (≤381)	31.07	21.00	38.25	13.02	11.65	12.25	39.90
	Q2 (381–448)	41.05	38.65	22.17	9.71	9.34	5.15	44.02
	Q3 (448–489)	23.00	19.00	14.25	4.85	4.03	3.17	31.35
	Q4 (>489)	116.16	112.00	89.75	19.88	17.87	18.37	143.64
All sources	Total	66.51	35.00	73.00	18.05	8.97	21.03	101.12
	95% CIs	56.06, 77.45	25.00, 50.00	54.50, 99.00	15.01, 21.40	7.55, 11.65	14.74, 27.41	85.70, 115.85
	Q1 (≤299)	44.74	23.00	56.00	25.55	15.09	29.48	67.94
	Q2 (299–407)	60.63	39.00	60.00	16.86	10.12	17.17	88.28
	Q3 (407–533.5)	76.34	32.15	96.50	16.52	7.59	22.80	118.42
	Q4 (>533.5)	84.53	54.00	117.00	13.26	7.02	19.40	120.60
**Proteins (g)**								
**Source**	**Range**	**MAE**	**MedAE**	**IQRAE**	**MAPE (%)**	**MedAPE (%)**	**IQRAPE (%)**	**RMSE**
Allrecipes.com	Total	6.36	4.10	6.60	43.15	20.65	40.76	9.49
	95% CIs	4.98, 7.81	2.85, 5.40	4.50, 8.72	30.08, 59.23	17.00, 28.42	30.27, 50.92	7.42, 11.45
	Q1 (≤12)	3.78	2.00	2.90	82.18	28.57	47.92	6.40
	Q2 (12–19)	3.41	3.20	4.67	22.26	20.03	33.53	4.44
	Q3 (19–28)	11.37	9.40	13.45	47.62	44.80	49.98	14.49
	Q4 (>28)	6.99	4.50	5.80	17.88	11.73	15.72	9.51
SNAPMe	Total	4.12	2.30	5.02	21.44	10.82	14.08	6.02
	95% CIs	3.18, 5.17	1.75, 3.10	2.70, 7.59	14.00, 33.11	9.16, 14.91	9.02, 26.71	4.65, 7.31
	Q1 (≤13.2)	2.07	0.80	1.36	34.20	10.42	13.92	3.91
	Q2 (13.2–22.6)	4.14	2.45	3.89	23.15	11.96	28.78	6.05
	Q3 (22.6–33.9)	5.20	3.05	6.37	19.01	11.82	20.25	7.16
	Q4 (>33.9)	5.11	3.70	5.90	9.35	8.62	11.52	6.52
Home-prepared	Total	3.13	2.28	3.50	11.32	9.29	9.65	4.01
	95% CIs	2.22, 4.20	1.50, 4.40	1.80, 4.80	8.36, 14.77	5.73, 14.08	4.77, 12.51	2.82, 5.22
	Q1 (≤18.2)	1.40	1.00	1.02	13.52	9.29	9.26	1.57
	Q2 (18.2–27)	2.15	1.50	0.96	8.78	5.64	3.32	2.86
	Q3 (27–38.8)	4.74	4.80	1.65	14.04	15.29	5.03	5.11
	Q4 (>38.8)	4.53	4.20	1.07	8.57	8.84	3.90	5.45
All sources	Total	5.09	2.90	5.44	30.83	15.00	26.77	7.75
	95% CIs	4.30, 5.94	2.30, 3.90	4.35, 7.00	23.28, 39.71	11.73, 17.06	18.13, 35.36	6.46, 9.04
	Q1 (≤13)	3.04	1.80	3.30	55.47	20.00	35.00	5.28
	Q2 (13–22)	4.70	2.55	4.50	25.43	13.65	34.02	7.27
	Q3 (22–33.3)	7.40	4.10	8.05	27.84	14.40	31.21	10.67
	Q4 (>33.3)	5.68	4.50	6.20	12.31	10.47	11.29	7.44
**Carbohydrates (g)**								
**Source**	**Range**	**MAE**	**MedAE**	**IQRAE**	**MAPE (%)**	**MedAPE (%)**	**IQRAPE (%)**	**RMSE**
Allrecipes.com	Total	7.81	5.25	9.90	42.54	24.95	37.26	12.00
	95% CIs	6.07, 9.67	3.00, 6.80	6.75, 13.70	28.19, 61.62	15.78, 29.29	30.10, 46.73	9.5, 14.78
	Q1 (≤12)	4.98	1.25	4.30	83.94	24.72	50.67	10.32
	Q2 (12–26)	7.04	6.70	4.10	37.56	32.31	23.33	8.58
	Q3 (26–41)	10.24	8.05	11.70	28.85	23.85	31.79	13.32
	Q4 (>41)	9.21	3.70	12.90	14.98	5.80	22.59	14.90
SNAPMe	Total	4.38	2.90	3.57	13.84	7.10	9.65	7.52
	95% CIs	3.14, 5.93	2.32, 3.70	2.72, 6.10	8.16, 22.30	5.35, 8.96	7.00, 13.11	4.43, 10.36
	Q1 (≤26)	4.18	2.60	2.87	28.08	12.02	15.23	8.57
	Q2 (26–40)	2.63	2.60	1.80	8.33	8.67	7.22	3.33
	Q3 (40–52.3)	4.78	2.70	4.93	10.64	5.77	9.73	8.90
	Q4 (>52.3)	5.78	4.20	4.50	6.83	6.56	3.89	7.59
Home-prepared	Total	6.48	3.70	7.59	17.98	12.68	19.74	9.17
	95% CIs	4.18, 9.28	1.60, 8.80	3.81, 11.50	11.82, 25.22	5.56, 23.53	9.07, 31.43	5.53, 12.71
	Q1 (≤33.5)	4.71	3.70	4.85	25.67	25.30	18.56	5.64
	Q2 (33.5–41.8)	2.64	1.35	1.07	6.62	3.80	2.81	4.01
	Q3 (41.8–46.4)	11.50	8.30	13.18	26.06	18.21	28.69	14.95
	Q4 (>550)	7.38	7.20	4.62	12.29	12.74	8.15	8.58
All sources	Total	6.34	3.70	6.67	28.50	12.22	24.91	10.15
	95% CIs	5.28, 7.51	2.90, 4.30	5.70, 8.30	20.70, 38.61	8.64, 14.79	20.04, 33.56	8.24, 12.07
	Q1 (≤ 17.9)	5.16	2.20	5.50	63.64	24.62	45.33	9.83
	Q2 (17.9–34.5)	5.27	3.70	6.60	20.66	13.53	23.75	7.10
	Q3 (34.5–46.2)	7.22	2.95	8.52	17.99	7.11	22.63	11.41
	Q4 (>46.2)	7.72	4.41	6.80	11.50	7.12	9.03	11.66
**Lipids (g)**								
**Source**	**Range**	**MAE**	**MedAE**	**IQRAE**	**MAPE (%)**	**MedAPE (%)**	**IQRAPE (%)**	**RMSE**
Allrecipes.com	Total	6.12	3.00	7.07	34.41	16.85	42.06	9.58
	95% CIs	4.68, 7.64	1.70, 4.60	5.00, 12.70	26.15, 43.62	10.00, 34.67	33.70, 55.48	7.57, 11.42
	Q1 (≤10)	2.43	1.30	2.50	36.80	26.00	44.29	4.19
	Q2 (10–19.5)	7.90	6.00	9.35	54.15	38.89	63.20	11.99
	Q3 (19.5–27.2)	6.16	4.60	7.65	25.25	17.78	30.65	8.31
	Q4 (>27.2)	8.23	4.05	13.67	22.18	8.61	42.32	11.91
SNAPMe	Total	4.58	1.50	4.28	21.64	12.21	20.21	8.42
	95% CIs	3.11, 6.29	1.10, 2.35	1.85, 8.22	15.88, 28.10	7.51, 17.38	13.73, 32.69	5.67, 10.96
	Q1 (≤11.2)	0.95	0.80	0.75	20.66	17.83	12.61	1.16
	Q2 (11.2–19.2)	3.19	1.27	1.78	19.32	9.12	12.46	6.98
	Q3 (19.2–30.8)	5.67	2.20	8.17	24.54	8.64	38.25	9.05
	Q4 (>30.8)	8.48	5.40	10.65	22.03	11.88	19.91	12.29
Home-prepared	Total	4.54	3.33	4.00	30.95	24.44	27.74	6.24
	95% CIs	2.98, 6.26	1.90, 5.00	2.10, 10.08	19.99, 44.89	15.47, 33.94	11.22, 43.13	4.04, 8.16
	Q1 (≤10.5)	3.58	1.90	3.23	40.79	27.14	31.13	6.46
	Q2 (10.5–15)	4.97	3.65	3.17	38.69	32.07	33.39	6.41
	Q3 (15–21.7)	3.65	3.81	2.19	18.23	17.64	11.92	4.00
	Q4 (>21.7)	6.10	5.40	6.55	24.46	23.03	30.20	7.52
All sources	Total	5.33	2.40	5.70	29.16	16.42	34.24	8.78
	95% CIs	4.39, 6.35	1.70, 3.00	4.35, 8.20	24.06, 34.73	11.88, 20.83	29.60, 41.93	7.31, 10.18
	Q1 (≤10.6)	2.06	1.00	1.80	32.35	20.00	33.45	3.91
	Q2 (10.6–18.7)	5.09	2.30	5.60	35.41	16.00	33.50	8.98
	Q3 (18.7–27.5)	5.93	4.45	7.77	26.25	17.78	32.95	8.16
	Q4 (>27.5)	8.26	4.40	13.20	22.64	10.18	32.88	12.07

**Note:** Values for each independent data source and for all combined data sources (global) are presented as overall results and by quartile. 95% CIs for all reported metrics were estimated via nonparametric bootstrap (10,000 resamples, dish-level).

**Table 6:** Inferential analysis of estimation errors across test cases and data sources.

Case	Variable	Kruskal–Wallis (H, p)	Pairwise Comparison	Mann–Whitney (U, p)	Holm $(p_{Holm}$)	Cliff’s δ
**1**	**Calories (kcal)**	**H = 7.863,** p**= 0.020**	Allrecipes.com vs. SNAPMe	U = 3553.5, p = 0.998	—	—
			**Allrecipes.com vs. Home-prepared**	**U = 1644.0,** p**= 0.005**	**0.018**	**Large**
			**SNAPMe vs. Home-prepared**	**U = 1219.5,** p**= 0.018**	**0.072**	**Large**
	Proteins (g)	H = 0.777, p = 0.678	—	—	—	—
	Carbohydrates (g)	H = 2.018, p = 0.365	—	—	—	—
	Lipids (g)	H = 1.205, p = 0.547	—	—	—	—
**2**	**Calories (kcal)**	**H = 9.043,** p**= 0.011**	Allrecipes.com vs. SNAPMe	U = 3367.5, p = 0.563	—	—
			**Allrecipes.com vs. Home-prepared**	**U = 1616.5,** p**= 0.008**	**0.031**	**Large**
			**SNAPMe vs. Home-prepared**	**U = 1286.5,** p**= 0.004**	**0.015**	**Large**
	Proteins (g)	H = 0.083, p = 0.960	—	—	—	—
	Carbohydrates (g)	H = 0.700, p = 0.705	—	—	—	—
	Lipids (g)	H = 0.208, p = 0.901	—	—	—	—
**3**	Calories (kcal)	H = 5.743, p = 0.057	—	—	—	—
	Proteins (g)	H = 1.936, p = 0.380	—	—	—	—
	Carbohydrates (g)	H = 1.428, p = 0.490	—	—	—	—
	Lipids (g)	H = 3.669, p = 0.160	—	—	—	—
4	Calories (kcal)	**H = 14.130,** p<**0.001**	**Allrecipes.com vs. SNAPMe**	**U = 4716.5,** p<**0.001**	**0.001**	**Large**
			Allrecipes.com vs. Home-prepared	U = 1477.5, p = 0.076	—	—
			SNAPMe vs. Home-prepared	U = 803.0, p = 0.328	—	—
	Proteins (g)	H = 2.005, p = 0.367	—	—	—	—
	Carbohydrates (g)	**H = 7.753,** p=**0.021**	**Allrecipes.com vs. SNAPMe**	**U = 4373.5,** p=**0.010**	**0.030**	**Small**
			Allrecipes.com vs. Home-prepared	U = 1176.0, p = 0.880	—	—
			SNAPMe vs. Home-prepared	U = 684.0, p = 0.053	—	—
	Lipids (g)	H = 3.229, p = 0.199	—	—	—	—

**Note:** *p*-values were adjusted using Holm’s sequential correction in two stages: first across pairwise comparisons among the three data sources (Allrecipes.com, SNAPMe, and Home-prepared) within each variable, and subsequently across variables (calories, proteins, lipids, and carbohydrates) within each test case to control the family-wise error rate. Only the final adjusted *p*-values ($p_{Holm}$) are reported. Values in bold indicate statistically significant differences ($p_{Holm}$ < 0.05). Cliff’s δ values range from −1 to +1, where |δ| ≈ 0.147, 0.333, and 0.474 correspond to small, medium, and large effect sizes, respectively.

## 4. Discussion

### 4.1. Results Interpretation

The results show that ChatGPT-5’s accuracy in estimating caloric and macronutrient content from meal photographs depends strongly on the amount and specificity of contextual information given. As shown in Table 2, Table 3 and Table 4, MAE, MAPE and RMSE decreased systematically across the three first experimental cases. Taking the total energy as an example, MAE fell from 123.03 kcal in Case 1 (image only) to 92.04 kcal in Case 2 (image with non-visual information) and further to 53.33 kcal in Case 3 (image with detailed ingredient list). The corresponding RMSE values decreased from 163.80 kcal to 122.48 kcal and 80.86 kcal, respectively. Likewise, the MAPE showed a substantial reduction, from 30.51% in Case 1 to 24.35% in Case 2 and 13.92% in Case 3. Both absolute and relative errors decreased as more contextual cues were provided.

When compared with Cases 1 and 2, Case 4 (summarized in Table 5) showed markedly lower errors, confirming that detailed ingredient information alone substantially improved estimation accuracy relative to image-only or limited-context inputs. However, when compared with Case 3, where the same textual information was provided but accompanied by the image, accuracy declined once the visual component was removed. For example, the total energy MAE increased from 53.33 kcal in Case 3 to 66.51 kcal in Case 4, while MAPE increased from 13.92% to 18.05% and RMSE from 80.86 kcal to 101.12 kcal. The qualitative analysis of ChatGPT-5’s responses revealed that, without access to the image, the model was sometimes uncertain about whether specific ingredients were raw or cooked, leading to greater variability in its estimations. In contrast, when visual cues were available, the model could infer cooking state and other preparation details, resulting in more accurate and consistent estimations across food items.

Scatter plots in Figure 1 further illustrate the trends. In Case 1, data points were widely dispersed around the identity line (particularly for lipids and proteins), indicating systematic over- and underestimation. As context was added (Cases 2 and 3), the scatter became progressively tighter and more symmetric, with points clustering closely around the y=x line, showing improved agreement between estimated and reference values. In Case 4, dispersion increased in comparison to Case 3. Data points deviated more noticeably from the identity line, reflecting a decline in estimation accuracy. Regarding proteins, Case 4 shows more overestimations than Case 3. This pattern likely arises from ChatGPT-5 interpreting certain protein sources (e.g., meats) as cooked rather than raw. Since such cooked items have a higher protein concentration per unit weight due to water loss, this misinterpretation can lead to inflated protein estimates and systematic overestimations.

Boxplots of absolute errors (Figure 2) corroborate the quantitative findings. MedAE and IQRAE decreased steadily from Case 1 to Case 3 for all variables and data sources. The Home-prepared dataset displayed consistently fewer outliers and, in most cases, narrower distributions, indicating greater consistency and less dispersion in estimation performance. In contrast, data from Allrecipes.com [20] and SNAPMe [11] exhibited broader variability, likely due to inconsistencies in recipe annotation, portion size, and photographic conditions. In all sources, greater errors (outliers) were observed in dishes served in bowls, which may relate to challenges in perceiving depth. Contrasting Case 4 with Case 3, error distributions widened again across most variables. MedAE increased slightly, and the interquartile ranges expanded, reflecting the model’s reduced accuracy when visual cues were removed.

To further investigate the differences in performance among data sources, an inferential analysis was conducted, as described in Section 2.5. As summarized in Table 6, significant differences in estimation errors among data sources were found primarily for caloric values. In Cases 1 and 2, the Allrecipes.com dataset produced significantly higher absolute errors compared to the Home-prepared dataset ($p_{Holm}\;$= 0.018 and 0.031, respectively). SNAPMe also differed from Home-prepared in both cases ($p_{Holm}$ = 0.072 and 0.015, respectively). These contrasts were associated with large effect sizes (Cliff’s δ > 0.474), indicating substantial disparities in performance across datasets. In contrast, macronutrient errors (proteins, carbohydrates, and lipids) did not show statistically significant differences across data sources in Cases 1–3.

Although total calories are computed from macronutrient content, the estimation errors for proteins, carbohydrates, and lipids do not necessarily vary in the same direction. Overestimation in one macronutrient (e.g., carbohydrates) may be offset by underestimation in another (e.g., lipids), resulting in a net caloric error that is smaller and more consistent across samples. This compensatory behavior can reduce variability in total energy estimates while keeping macronutrient-specific errors high and uneven, which diminishes the likelihood of detecting statistically significant differences for individual nutrients. Consequently, the overall caloric estimation becomes a more stable indicator of performance differences among data sources.

In Case 3, no significant differences were detected between sources, since the inclusion of detailed ingredient lists and quantities homogenized the input information and reduced between-source variability. Under these conditions, ChatGPT-5 achieved uniformly low errors across all datasets, indicating that sufficient contextual detail allows the model to reach a consistent estimation performance regardless of the data source.

Unlike Case 3, Case 4 exhibited statistically significant differences for calories and carbohydrates, with Allrecipes.com showing higher errors than SNAPMe ($p_{Holm}$ = 0.001 and 0.030, correspondingly). These results suggest that, when visual information was removed, variability between datasets increased again, particularly affecting energy and carbohydrate estimations. In Case 4, ChatGPT-5 appeared more sensitive to the structure of textual descriptions, as visual information was unavailable. In particular, the sometimes more narrative format of Allrecipes.com seems to make ingredient interpretation more ambiguous, leading to higher estimation errors compared with the more organized entries from SNAPMe.

The relationship between dish complexity (measured as the number of ingredients) and the absolute caloric estimation error was analyzed through Pearson’s correlation coefficient and linear regression across the experimental cases and data sources. Overall, weak positive correlations (r≤ 0.37) were observed in all cases (Figure 3), indicating that the estimation error tended to increase slightly as the number of ingredients grew, but the effect remained modest. This suggests that while greater ingredient diversity may add visual and contextual complexity, it does not substantially compromise ChatGPT-5’s ability to generate nutritional estimates.

Across test cases, the correlation strength varied mildly: the lowest values were found in Case 2, reflecting the stabilizing effect of non-visual contextual information, while Case 3 showed higher coefficients, likely because detailed ingredient lists in combination with food images exposed residual inconsistencies in complex dishes. Despite these slight variations, the regression slopes remained low in all cases, and the confidence ellipses progressively narrowed from Case 1 to Case 3, consistent with the overall reduction in MAE and RMSE. These results confirm that as contextual information becomes richer, ChatGPT-5’s estimates grow more reliable. In Case 4, correlations decreased slightly when compared with Case 3. When analyzed by data source, the Home-prepared dataset exhibited the weakest correlations, denoting consistent performance even for multi-ingredient meals. In contrast, Web-based and SNAPMe datasets displayed broader variability and slightly higher regression slopes.

### 4.2. Comparison with Related Work

The findings of this study confirm the potential of ChatGPT-5 for dietary assessment from meal photographs, especially when supported by complementary information. This aligns with prior work by Zheng [4] and Ege [9], who concluded that AI models achieve higher accuracy when provided with contextual information such as ingredients or cooking methods. In particular, Ege [9] showed that integrating such variables reduces relative error and strengthens correlations with reference values.

Notably, although ChatGPT-5 is not specifically designed for nutritional assessment, its performance was comparable to that of models explicitly trained for this purpose. Fang [7] reported an average error of 209 kcal, whereas ChatGPT-5 achieved a MAE of 123.03 kcal using images alone. When full contextual information was incorporated, the error decreased to 53.33 kcal, approaching the 37.9 kcal reported by Wang [10] with a more advanced architecture.

In contrast to the findings of Sultana [6] and Wang [10], who reported major difficulties for AI models in assessing multi-ingredient dishes, our study identified only a weak positive correlation between the number of ingredients and absolute error. This indicates that dish complexity, in terms of component count, is not a critical determinant of ChatGPT-5’s estimations. Rather, elements such as image quality, level of detail, and the inclusion of reference objects seem to have a stronger influence on accuracy, consistent with observations by Akpa [8] and Ege [9].

When contrasted with the recent evaluation of ChatGPT-4 by O’Hara et al. [32], our findings provide complementary evidence. Their study assessed 114 standardized meal photographs from the Irish National Adult Nutrition Survey (NANS), focusing on the model’s ability to identify foods and estimate nutrient content. ChatGPT-4 achieved high accuracy in food recognition (accuracy 93%, recall 84.6%, harmonic mean 88.6%). For nutrient estimation, correlation with reference values ranged from weak (0.29) to strong (0.83), indicating that the model’s performance varied depending on the nutrient considered. Nutrient underestimation was frequent, with a MAPE of 26.9% and marked discrepancies for certain micronutrients, such as vitamin D (−100%) and potassium (−49.5%). Our framework, which incorporated contextual information, yielded substantially lower errors for energy and macronutrients, suggesting that enriched prompts and detailed input data can mitigate systematic underestimations observed in baseline evaluations.

While the study by O’Hara et al. [32] shows the feasibility of applying ChatGPT-4 to dietary assessment, it was constrained by reliance on a single, relatively homogeneous dataset focused on Irish meal types. In contrast, the present work incorporates a more heterogeneous database, combining meals prepared and annotated by nutritionists, images from the SNAPMe repository [11], and recipe photographs sourced from the web. In addition, the latest version of ChatGPT was utilized, and the model was systematically evaluated under different levels of contextual information. Overall, the approach of this study not only allows for a more realistic and culturally diverse assessment but also offers a clearer understanding of how non-visual information contributes to the model’s accuracy.

An important observation concerns the influence of data source on estimation accuracy. Photographs of Home-prepared meals, annotated with directly measured ingredients and standardized equivalence tables, consistently showed lower errors, whereas external sources (i.e., Allrecipes.com and SNAPMe) displayed greater variability. This supports Larke’s [11] observation that databases built by individuals without formal training in nutrition may contain inaccuracies in portion sizes, ingredient descriptions, or nutritional values.

Although the employed dataset underwent a curation process to remove inconsistent entries and standardize nutritional information, residual inaccuracies cannot be fully excluded. The higher errors observed in external sources may partly reflect inaccuracies in the ground truth. During database development, several records had to be discarded or corrected due to clearly erroneous annotations, revealing issues in data collection. In the case of SNAPMe, limitations of the data-entry application and user inexperience often resulted in inaccurate quantities or the inclusion of false ingredients. Likewise, nutritional information obtained from Allrecipes.com frequently failed to match the portion sizes or ingredients displayed in the images. In addition, user-uploaded web photographs exhibited greater heterogeneity in angle, lighting, and overall quality, further contributing to variability in estimation accuracy.

### 4.3. Practical Application of ChatGPT

As a complementary outcome of this work and to exemplify the versatility and ease of use of ChatGPT-5, a customized version of the model was developed, tailored for dietary assessment from dish photographs. The process is straightforward and requires only simple instructions in natural language. Details of its creation and an illustrative example are provided in Appendix C.

Building upon the experience gained throughout this study, the customized model is designed to prompt users for all available contextual information, such as ingredient lists, portion sizes, and preparation methods. Yet, it retains the capability to generate estimates in the absence of any accompanying metadata, i.e., when only an image is provided.

### 4.4. Open Issues

- Higher estimation errors were consistently observed for dishes served in bowls, likely reflecting ChatGPT-5’s limited ability to infer depth and volume from monocular images. Future work could evaluate the effectiveness of including a vertically oriented reference object placed beside the dish to improve volumetric estimation.
- Since ChatGPT-5 is a pre-trained, closed-weight model, conventional validation schemes such as holdout test sets or k-fold cross-validation cannot be directly applied. The model’s parameters are fixed and not updated during use, so its outputs reflect generalization learned during large-scale pretraining rather than task-specific adaptation. Nevertheless, such validation procedures would be highly relevant in the context of fine-tuned or customized derivatives of ChatGPT-5, as described in Appendix C. For these tailored versions, systematic partitioning of annotated data into training, validation, and test sets (or stratified k-fold cross-validation) would enable quantitative assessment of generalizability and overfitting. Furthermore, iterative prompt engineering and supervised fine-tuning on domain-specific image–nutrient pairs could improve accuracy, reduce variance across food types, and enhance reliability for practical dietary assessment applications.
- A major limitation for future research is the absence of broad, high-quality, and systematically annotated datasets linking food images to verified nutritional composition. The development of such a database, curated by nutrition professionals, would provide a reliable foundation for both model evaluation and fine-tuning. At Universidad Europea del Atlántico, such an initiative is being considered, involving active participation of students in Human Nutrition and Dietetics or related disciplines. Leveraging their technical training and the institution’s multicultural environment would ensure both the accuracy and diversity of the dataset, enhancing its representativeness for real-world dietary applications.
- Future research should include direct benchmarking of ChatGPT-5’s nutritional estimations against both traditional computer vision models and professional dietitian assessments. Such head-to-head evaluations would provide a clearer understanding of the model’s relative strengths and limitations, quantify its added value beyond established analytical frameworks, and support evidence-based assessment of its potential integration into clinical nutrition and dietary evaluation workflows.

## 5. Conclusions

The main objective of this study was to evaluate the accuracy of ChatGPT-5 in estimating caloric content and macronutrient distribution from meal photographs. A total of 195 dishes were analyzed. The imagery and nutritional information were obtained from three sources: two external datasets (Allrecipes.com [20] and SNAPMe [11]) and one internally developed by nutritionists. Four scenarios with varying types and levels of contextual information were tested, showing that accuracy improved consistently when visual inputs were complemented with additional non-visual cues and detailed ingredient descriptions. However, the decline in accuracy observed when the image was omitted, despite the inclusion of detailed ingredient information, indicates that visual cues contribute markedly to estimation performance, and that the observed improvements cannot be attributed solely to arithmetic derived from ingredient lists. Dish complexity, measured by the number of ingredients, showed a weak positive correlation, suggesting that complexity alone is not decisive.

The lowest errors were consistently observed in self-prepared and annotated meals, whereas external sources exhibited greater variability. This finding underscores the limitations of relying on datasets created without nutritional expertise and the need for professionally curated image-based resources. Developing robust and multicultural databases under the supervision of nutrition professionals is, therefore, essential to ensure accuracy, reduce variability, and make the evaluation of AI models (such as ChatGPT-5) in dietary assessment fairer and more representative.

In summary, this study provides novel evidence supporting a potential complementary role of ChatGPT-5 within clinical nutrition workflows. However, confirmatory head-to-head evaluations against registered dietitians and validated assessment tools are still needed before clinical adoption. Although limitations persist, particularly with external data sources, the findings emphasize its potential as a complementary tool for dietary evaluation, with promising applications in both clinical practice and nutrition research.

## Figures and Tables

**Table 1:** Key considerations from the literature.

Topic	References	Considerations
**Model Performance**	Sultana et al. [6], Akpa et al. [8], Ege et al. [9].	Use of reference objects (e.g., cutlery and chopsticks) to improve food volume and depth estimation.
	Zheng et al. [4], Fang et al. [7], Wang et al. [10].	Difficulty in identifying ingredients in complex dishes. Incorporation of contextual information (e.g., type and content of fat and sweeteners) is recommended.
	Akpa et al. [8], Ege et al. [9], Wang et al. [10].	Estimates affected by camera angle, lighting, occlusions, tableware color, and image resolution.
**Evaluation metrics and methods**	Shonkoff et al. [5].	Absolute and relative error, Root Mean Square Error (RMSE).
	Ege et al. [9].	Pearson correlation coefficient and regression analysis.
	Wang et al. [10].	Mean Absolute Error (MAE), Mean Absolute Percentage Error (MAPE), and visual analysis using scatter plots.
**Databases**	Zheng et al. [4], Sultana et al. [6], Ege et al. [9].	Use of images from social media and websites to represent diverse cuisines, including real-life scenarios and multi-ingredient dishes.
	Larke et al. [11].	SNAPMe: publicly available database of food photographs with portion size and nutritional annotations.

**Figure A1:**
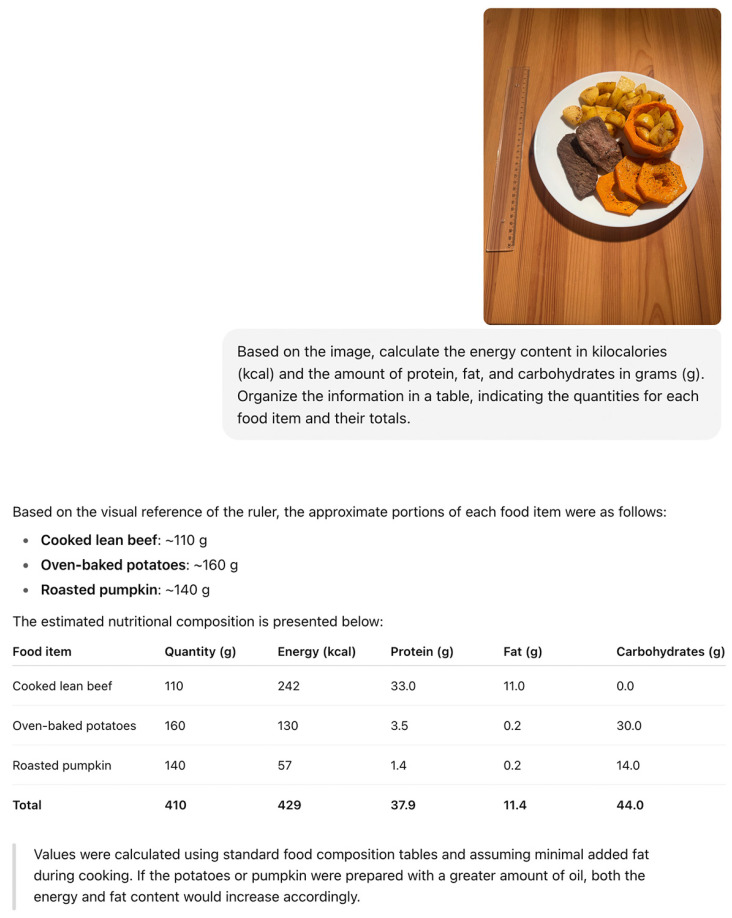
Use of ChatGPT-5 for test case 1 (image only).

**Figure A2:**
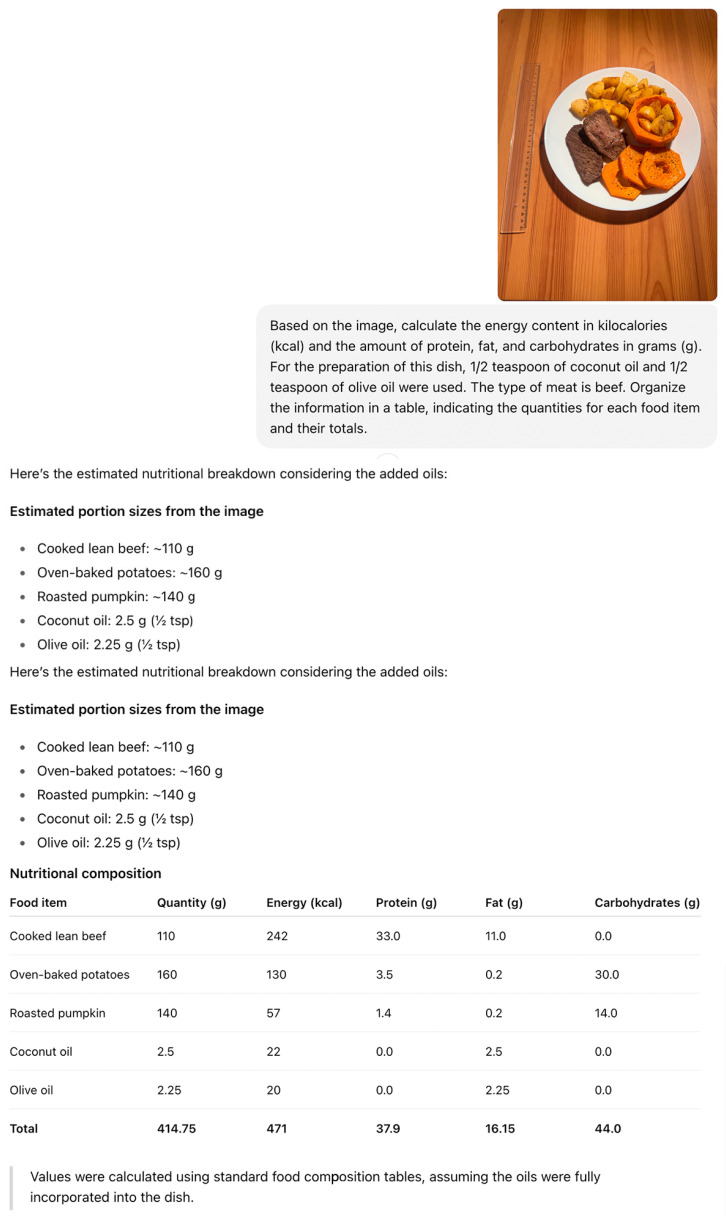
Use of ChatGPT-5 for test case 2 (image with non-visual data).

**Figure A3:**
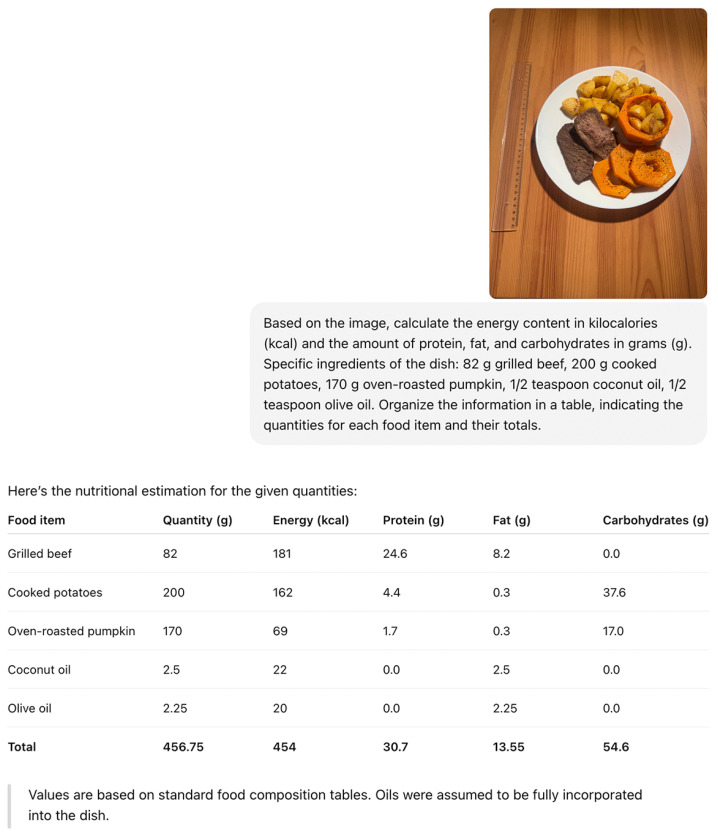
Use of ChatGPT-5 for test case 3 (image with detailed ingredient information).

**Figure A4:**
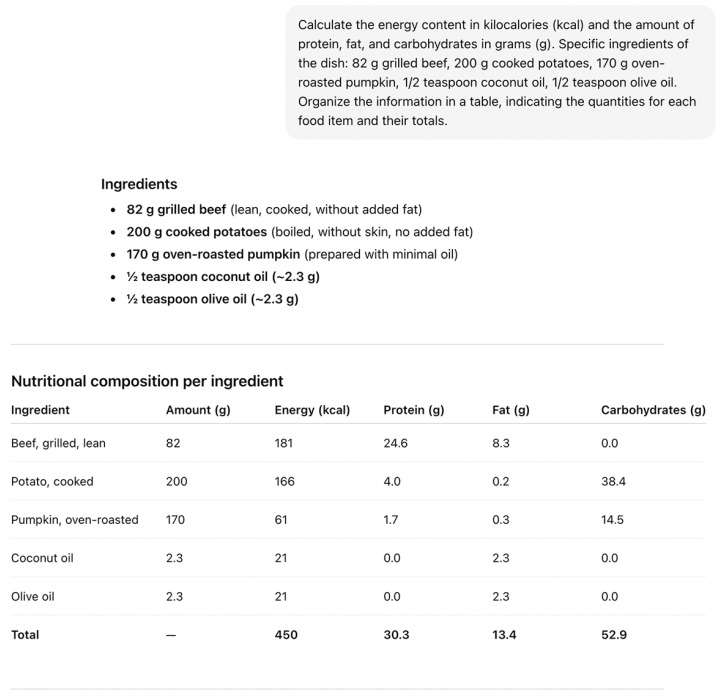
Use of ChatGPT-5 for test case 4 (detailed ingredient information only).

**Figure A5:**
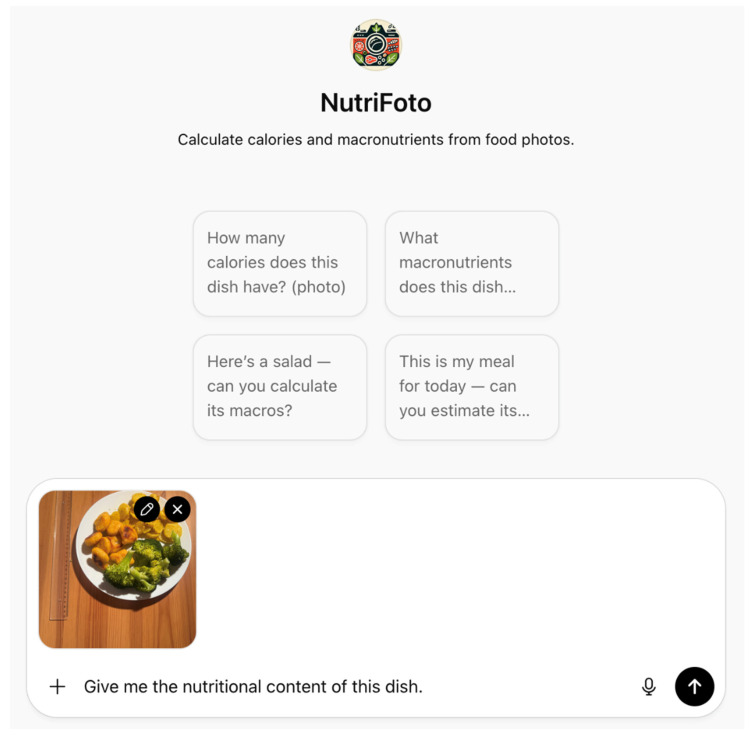
Input interface of the custom GPT *NutriFoto*.

**Figure A6:**
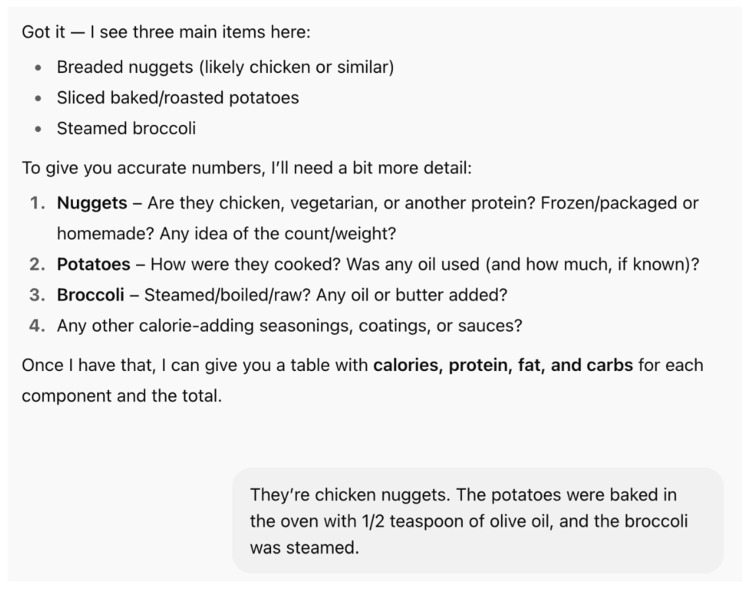
*NutriFoto* requesting additional information.

**Figure A7:**
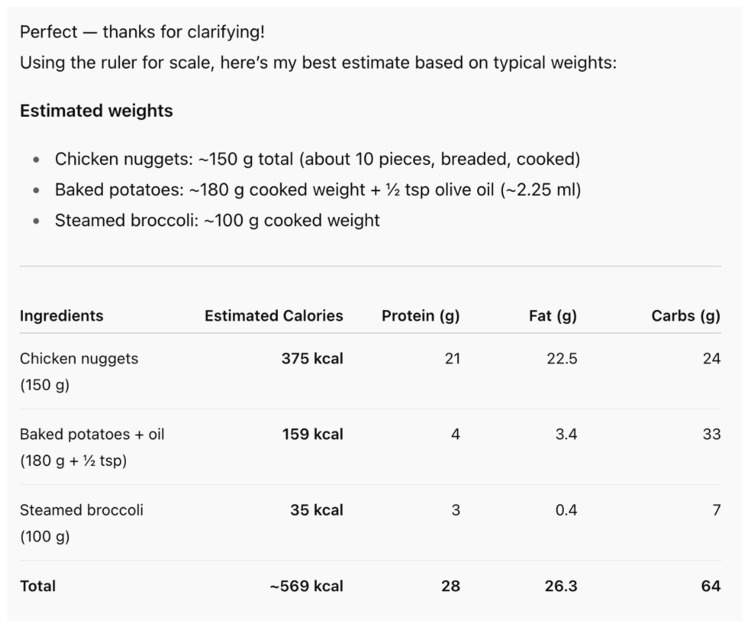
Result of the nutritional evaluation from *NutriFoto*.

## Data Availability

The original contributions presented in the study are included in the article; further inquiries can be directed to the corresponding authors.

## Abbreviations

The following abbreviations are used in this manuscript:

AI	Artificial Intelligence
API	Application Programming Interface
CI	Confidence Interval
DL	Deep Learning
GPT	Generative Pre-trained Transformer
IQR	Interquartile Range
IQRAE	IQR of Absolute Errors
IQRAPE	IQR of Absolute Percentage Errors
MAE	Mean Absolute Error
MedAE	Median Absolute Error
MAPE	Mean Absolute Percentage Error
MedAPE	Median Absolute Percentage Error
NANS	National Adult Nutrition Survey
RMSE	Root Mean Square Error
SMAE	Mexican System of Food Equivalents
SNAPMe	Surveying Nutrient Assessment with Photographs of Meals
USDA	U.S. Department of Agriculture

## Appendix A

The database for this study comprises photographs, reference nutritional values, and ingredient lists for 195 dishes. Table A1 illustrates its structure, providing an example of the sources from which the data were collected, namely, Allrecipes.com [20], SNAPMe [11], and Home-prepared.

**Table A1:** Database organization.

**Image**	**Ingredients**	**Energy (kcal)**	**Proteins (g)**	**Lipids (g)**	**Carbohydrates (g)**
**** Allrecipes.com [20]	¼ cup vegetable oil; 3 corn tortillas (6 inches) cut into strips; ¾ dried pasilla chile (seeded); ½ tomato (seeded and chopped); ⅛ large onion (chopped); ¼ garlic clove; a pinch of dried oregano (≈1/16 teaspoon); ¼ teaspoon vegetable oil; 1 cup of water; 1 teaspoon chicken bouillon granules; ¼ sprig fresh parsley; ¼ avocado (peeled, pitted, and diced); 3 tablespoons cotija cheese (diced); ½ tablespoon crème fraîche (or Mexican crema).	944	52	80	13
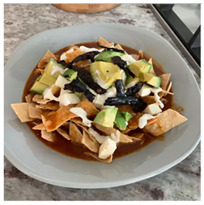
**** SNAPMe [11]	120 g breaded chicken fillet; 15 g honey mustard dip; 180 g potato, French fries (baked); 13.5 g olive oil; 66.6 g French bread; 5 g salted butter stick.	960	29	55	95
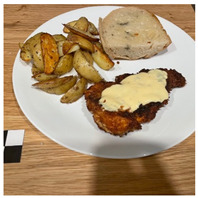
**** Home-prepared	82 g grilled beef; 200 g boiled potatoes; 170 g baked pumpkin; ½ teaspoon coconut oil; ½ teaspoon olive oil.	459	38.84	11.00	58.60
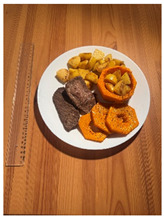

## Appendix B

This appendix presents an example of the application of ChatGPT-5 to each case study described in Section 2.6. The Home-prepared dish from Appendix A is used as the basis.

## Appendix C

The observations derived from the analysis in this study lay the foundation for the development of *NutriFoto*, a customized Generative Pre-trained Transformer (GPT) designed for dietary assessment based on meal photographs. ChatGPT permits the creation of personalized versions of the GPT model through a user-friendly interface [33]. The steps for generating a customized GPT are described below, followed by the specific instructions used to develop *NutriFoto*. Finally, an example of its application is presented.

### Appendix C.1. Steps to Create a Customized GPT

1. Access to the creation function. Log into chat.openai.com with a Plus account. In the side panel, select the “Explore GPTs” option, then click “Create a GPT” to launch the setup assistant.
2. Creation assistant. The system guides the user through a step-by-step wizard, in which the purpose of the GPT is described. Based on this information, the assistant generates an initial configuration that can be modified.
3. Customization of instructions. The user may edit the system instructions, which define the GPT’s behavior. These instructions can include, for example, the tone of the responses (formal, colloquial, etc.) and the area of specialization (e.g., nutrition).
4. Inclusion of tools and resources (optional). The GPT’s functionality can be expanded through the incorporation of additional resources, such as files or external links.
5. Model testing. Before finalizing the process, the GPT’s behavior can be tested within the platform. This stage allows for adjustments to the instructions or the correction of any issues identified during testing.
6. Publication and use. Finally, the GPT can be private (for exclusive use by the creator), shared via link (accessible only to recipients), or public (available in the GPT gallery for all users). A distinctive name, brief description, and logo may also be added.

### Appendix C.2. Instructions Used to Create NutriFoto

The following instructions correspond to Step 3 of Appendix C.1, where the GPT’s behavior is defined and adapted to the intended purpose.

- This GPT is a nutrition-specialized assistant designed to calculate the calories and macronutrients (protein, lipids, and carbohydrates) of dishes based on photographs uploaded by the user. Its objective is to provide an estimate based on image analysis, with optional support from additional user-provided data.
- The photograph is the only essential requirement. The assistant should greet the user, request the image, and suggest including a reference object (such as a ruler, spoon, fork, or chopsticks) to improve accuracy. If no reference object is present, it will proceed with the image and inform the user that the estimate may be less accurate.
- Before providing any estimate, the assistant should gather as much contextual information as possible, in the following order of priority: type and amount of fats, type of meat, type and content of sweeteners, fat content of dairy products, cooking method, individual ingredients (if the user can provide them), and the name of the dish (if known). If the image contains wrapped or stuffed foods, it should suggest (only when feasible) cutting them in half to view the contents.
- Once the information is collected, the assistant will present the results in a clear table with the fields: ingredients (if known), calories, protein, lipids, and carbohydrates. The table will be accompanied by a brief interpretation written in accessible language for the user. The tone should be warm, educational, and non-judgmental.

### Appendix C.3. Illustrative Application of NutriFoto

This section provides an illustrative example of *NutriFoto*, showing how the tool processes a meal photograph and generates nutritional estimates based on both visual and contextual information. Figure A5 displays the input interface of *NutriFoto*. Figure A6 then presents the model’s initial response, where contextual information is requested from the user. Once these data are supplied, *NutriFoto* generates a structured output including estimated caloric content and macronutrient distribution, as shown in Figure A7.

In the illustrative example, the reference values were 557 kcal, 23.5 g of protein, 64.6 g of carbohydrates, and 23 g of lipids. To refine the estimation, additional essential information was provided, including type and amount of fats, type of meat, and cooking method.

*NutriFoto* has been made publicly available and can be accessed online at: https://chatgpt.com/g/g-680616e7ec848191aebe3495da3d5587-nutrifoto (accessed on 13 November 2025).
